# Graduate Student Evolutionary Algorithm: A Novel Metaheuristic Algorithm for 3D UAV and Robot Path Planning

**DOI:** 10.3390/biomimetics10090616

**Published:** 2025-09-12

**Authors:** Xiaoxuan Liu, Shaobo Li, Yongming Wu, Zijun Fu

**Affiliations:** 1 State Key Laboratory of Public Big Data, Guizhou University, Guiyang 550025, China; gs.xxliu21@gzu.edu.cn; 2 Department of Information Engineering, Bijie Industry Polytechnic College, Bijie 551700, China; 3 Guizhou Institute of Technology, Guiyang 550025, China; 4 College of Information Science, Guizhou University of Finance and Economics, Guiyang 550025, China; wu20811055@163.com; 5 College of Computer Science and Technology, Guizhou University, Guiyang 550025, China; zijunfu@163.com

**Keywords:** artificial intelligence, numerical optimization, 3D UAV path planning, robot path planning

## Abstract

In recent years, numerical optimization, UAVs, and robot path planning have become hot research topics. Solving these fundamental artificial intelligence problems is crucial for further advancements. However, traditional methods struggle with complex nonlinear problems, prompting researchers to explore intelligent optimization algorithms. Existing approaches, however, still suffer from slow convergence, low accuracy, and poor robustness. Inspired by graduate students’ daily behavior, this paper proposes a novel intelligent optimization algorithm, the Graduate Student Evolutionary Algorithm (GSEA). By simulating key processes such as searching for research directions and concentrating on studies, a mathematical model of GSEA is established. The algorithm’s convergence behavior is analyzed qualitatively, and its performance is evaluated against competitive algorithms on the CEC2017 and CEC2022 test sets. Statistical tests confirm GSEA’s effectiveness and robustness. To further validate its practical applicability, GSEA is applied to UAV and robot path planning problems, with experimental results demonstrating its superiority in solving real-world optimization challenges.

## 1. Introduction

Path planning, as an essential technique, plays a crucial role in various domains. Its core objective is to find the optimal path from the starting point to the endpoint under various constraints. Whether a UAV performs missions or a robot accomplishes tasks, path-planning techniques are used to find the optimal path. An excellent path-planning algorithm can help efficiently solve various complex navigation problems. Path planning can help optimize traffic flow, reduce congestion, improve road utilization, and provide a good travel experience in urban traffic management [1]. Path planning can reduce transportation costs, improve the efficiency of goods delivery, and optimize the warehousing and distribution process in logistics and supply chain management [2]. Path planning can reduce transportation costs, improve the efficiency of goods delivery, and optimize the warehousing and distribution process. Path planning is the core technology in UAV flight [3] and robot navigation [4] to realize intelligent flight and navigation, ensuring that UAVs or robots can reach their destinations safely and efficiently. Therefore, there is an urgent need for each field to explore path-planning technology suitable for reducing expenditure costs and improving work efficiency. Consequently, studying path-planning algorithms has increasingly become a hot research topic.

Path-planning algorithms are methods to find optimal paths in a graph or network abstracted from a realistic scenario [5]. We can classify existing path-planning algorithms into two categories: traditional path-planning algorithms and intelligent optimization algorithms. Traditional path planning algorithms mainly include the artificial potential field method [6], the A* algorithm [7], Dijkstra’s algorithm [8], the Probabilistic Roadmap (PRM) algorithm [9], the rapidly expanding random tree (RRT) algorithm [10], and so on. Although previous researchers have developed many path-planning algorithms, and their mathematical theory has matured significantly, when applied to real path-planning problems, they often need help to avoid falling into local optima and fail to find the optimal path quickly.

**Figure 1:**
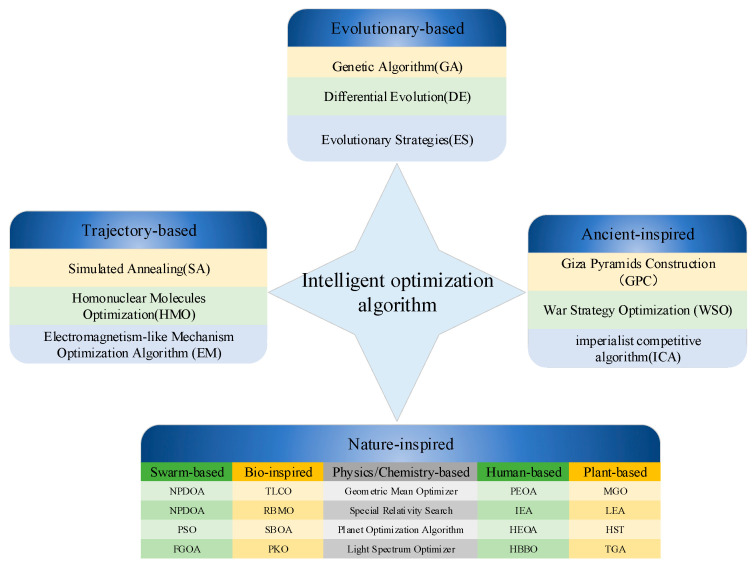
Classification of intelligent optimization algorithms.

In recent years, with the development of stochastic search theory, many emerging intelligent optimization algorithms have been developed to overcome this problem and have been successfully applied to path planning problems with promising results. The main ones are the traditional particle swarm algorithm [11], the genetic algorithm [12], the newly proposed alpha evolution [13], escape algorithm [14], and K-means optimizer [15]. Compared with traditional path planning algorithms, intelligent optimization algorithms retain the characteristics of good global exploration performance characteristics in Monte Carlo methods while possessing strong local exploitation abilities in heuristic methods. Therefore, they are not only used in path planning but are also widely applied in various fields [16], including computer science [17], optimal scheduling problems, transportation problems [18], combinatorial optimization problems, energy [19], health, stock market, economy, industry, structural and civil engineering, intelligent city planning, automated factories, aviation, operations research, and other fields. However, it is essential to note that different intelligent optimization algorithms have different characteristics and scopes of application. In Figure 1, we classify intelligent optimization algorithms based on their formation principles.

An intelligent optimization algorithm is inspired by human intelligence, the social nature of biological groups, or the laws of natural phenomena [20]. It optimizes the objective function by finding the optimal or near-optimal solution in the solution space. It is better to avoid falling into local optima than traditional local search methods. Moreover, it is usually insensitive to the problem’s initial conditions and parameter settings, which makes the algorithm perform well in various situations. It has better flexibility, but the NO Free Lunch theorem shows no optimization algorithm is effective for all optimization problems [21]. Researchers need to modify and adapt the algorithm to different optimization problems according to the characteristics of the problem to be optimized. Based on the previous research and the motivation of the NFL theorem, this paper proposes a new meta-heuristic algorithm named Graduate Student Evolutionary Optimization Algorithm (GSEA) based on the current behavior of graduate students, such as learning. In GSEA, we simulate the behaviors of current graduate students, such as study, life, and future further study or work to construct this algorithm for solving numerical optimization and path planning problems. The following summarizes several contributions made in this paper:

1. A review of the existing relevant literature on the subject and a discussion of its strengths and weaknesses.
2. Proposed a novel Graduate Student Evolutionary Algorithm (GSEA).
3. Compare the performance of the proposed algorithm with 11 new classical algorithms on 42 test functions in two test sets, CEC2017 and CEC2022.
4. The algorithms proposed in this paper are utilized to solve real-life path planning problems, such as UAV path planning and robot path planning problems.

The next part of the paper is organized as follows: Section 2 reviews existing techniques on path planning aspects. Section 3 describes the inspiration and the mathematical model of GSEA proposed in this paper. In Section 4, we perform numerical experiments on the algorithm proposed in this paper and test its performance. In Section 5, we apply the GSEA algorithm to real-world problems, i.e., UAV and robot path planning. A comprehensive and robust analysis of the algorithm is provided. Finally, in Section 6, we summarize and look at the outlook.

## 2. Literature Review

In this section, we review the literature related to this paper. With the rapid development of UAVs, robots, and other industries, there is an urgent need for an excellent path-planning algorithm.

In 2008, Chen Mou et al. proposed a three-dimensional real-time planning method for unmanned aerial vehicles based on an improved ant algorithm [22]. This algorithm reduces the possibility of local optimization of the original algorithm and speeds up the search for the global optimal solution, enabling dynamic planning schemes for UCAVs. Gene Eu Jan et al. introduced a higher geometric maze routing algorithm [23]. The free workspace is obtained by virtually expanding obstacles in the image, and the eight-quadrant maze routing algorithm is applied to find the best collision-free path with linear time and space complexity. In 2009, Haibin Duan et al. proposed an improved method of the IWD optimization algorithm for single-engine UCAV smoothing trajectory planning in complex combat environments [24]. They also introduced κ trajectory for smoothing UCAV trajectories, offering flexibility in dynamic environments and emergent threats. M.A. Porta Garcia et al. introduced a new method for solving mobile robot path-planning problems based on a simple ant colony optimization meta-heuristic [25]. This method incorporates the effect of existing distances between source and target nodes in the decision-making process. It endows ants with memetic properties to remember visited nodes, achieving a 10% speedup and supporting static and dynamic obstacle avoidance. In 2010, Rahul Kala et al. combined the A* algorithm and fuzzy reasoning to solve robot path planning problems efficiently [26]. Genetic algorithms optimize fuzzy parameters, ensuring the shortest path in finite time while considering obstacle distance, total path length, and turn sharpness. Chunfang Xu et al. proposed an improved ABC optimization algorithm based on chaos theory for UCAV path planning in various combat environments [27]. They provided detailed implementation processes and experimental comparisons, verifying the proposed method’s feasibility, effectiveness, and robustness. In 2011, Michaël Soulignac et al. proposed the sliding wavefront expansion method [28], guaranteeing the existence of paths with arbitrary accuracy through appropriate cost functions and continuous optimization techniques. Paths’ effectiveness and global optimality were theoretically proved, supported by realistic environment simulation results. Wang Chiung-Ying et al. introduced a scenario-aware path-planning mechanism based on Spatial Conceptual Maps (SCM) and Genetic Algorithms (GA) [29]. Using the SCM model to represent the surrounding environment and GA to plan optimal paths, this method finds paths tailored to individual user needs. In 2012, Y. Volkan Pehlivanoglu proposed the Multifrequency Vibrational Genetic Algorithm, improving the initialization phase and introducing periodic mutation strategies [30]. This algorithm was successfully applied to sinusoidal and urban terrain models, significantly reducing computation time. Rahul Kala proposed a co-evolutionary genetic programming method for solving multi-robot motion planning problems [31]. Operating at two levels, this method effectively solves motion planning problems in hybrid scenarios through linear genetic programming representation and genetic algorithm selection. In 2013, Mansoor Davoodi et al. proposed a genetic algorithm with an NSGA-II framework for solving decisive single-objective and a multi-objective path planning problem [32]. The proposed algorithm innovatively proposes a path refinement operator on top of the standard genetic operator, which enhances the algorithm’s exploration capability. The algorithm can find the optimal solution in complex path planning problems such as narrow channels and cluttered spaces. Fu Yangguang et al. proposed a hybrid differential evolution algorithm and quantum particle swarm optimization algorithm, which combines the DE algorithm and QPSO algorithm for UAV maritime route planning [33], and the proposed algorithm can generate high-quality paths faster than the real-valued genetic algorithms and standard particle swarm algorithms. 

In 2014, Zhu Weiren et al. proposed an improved biogeography-based optimization algorithm for solving optimization problems in the path planning process [34], which obtains a new search mechanism by employing chaos theory and predator–prey concepts, which can plan higher quality flights for unmanned combat aerial vehicles in comparison with the basic BBO algorithm, CBBO, and PPBBO paths. Asl Ali Noormohammadi et al. proposed using the asexual reproduction optimization (ARO) technique to plan optimal paths for multiple robots in a leader–follower structure in the presence of obstacles [35]. A novel mobile robot formation controller based on the potential field approach was also proposed, which was experimentally demonstrated to have faster convergence speed and higher performance than algorithms such as simulated annealing. Convergence speed and higher performance than algorithms such as simulated annealing, which is more suitable for real-time control applications. In 2015, Zhu Zexuan et al. proposed a multi-objective memory algorithm (MOMA) for path planning of wheeled robots [36]. Firstly, based on the traditional multi-objective genetic algorithm, elite non-distributive sorting and decomposition strategies were used to optimize the path length and smoothness. Secondly, a new path coding scheme, path refinement, and specific evolutionary operators were also introduced to improve the algorithm’s search capability and ensure the safety of candidate paths obtained in complex environments. Mo Hongwei et al. proposed a new method for global path planning in static environments combining BBO, PSO and Approximate Voronoi Boundary Networks (AVBN) [37], which utilizes the PSO’s position updating strategy to increase the diversity of the populations in the BBO algorithm, and then utilizes the resulting BPSO algorithm to perform path planning on the path network obtained through AVBN modelling. The results of simulation experiments show that this algorithm obtains competitive results. In 2016, since path length is not our only optimization criterion in many practical applications, Oral Tugcem et al. proposed a novel multi-objective incremental algorithm based on the D*lite algorithm-Multi-objective D*lite [38], which by introducing multi-objectivity into the solution space makes it usable for optimizing multiple objectives in dynamic and partially observable search domains. In addition, this algorithm can be run in a multi-agent environment, where each agent can autonomously execute its planner and cooperate with other agents to reach the goal location. Li Jiangiang et al. proposed a hybrid path planning algorithm that uses a genetic algorithm for global path planning and local rolling optimization for continuously optimizing the results of the genetic algorithm to obtain less costly paths [39], and this algorithm can run in real-time to support UAV/UHV systems. In 2017, Tong Junhua et al. developed a greedy genetic algorithm for path optimization by combining greedy algorithm (GRA) and genetic algorithm (GA) [40], significantly reducing the optimization time and improving the optimization efficiency. Since not all environments are well-organized during path planning, Thi Thoa Mac et al. proposed a novel hierarchical global path planning method for mobile robots in cluttered environments [41], which applies the accelerated updating method of the Pareto dominance principle to PSO in order to solve the robotic path planning problem. The path length of the robot, as well as the path smoothness, are considered globally.

In 2018, Wu Xiande et al. fused PSO, Metropolis Criterion, and Rauch–Tung–Striebel to propose a method to solve the path planning problem [42]. This method utilizes the Rauch–Tung–Striebel smoother to eliminate irregular errors in the PSO update position, resulting in smoother generated paths. Using Metropolis Criterion as an acceptance policy increases the likelihood of jumping out of local optima. Yong Ma et al. introduced a path-planning algorithm for solving multi-objective [43], multi-constraint nonlinear optimization models. This algorithm integrates an intelligent search method module, a dynamic update module for profiles and leaders, and a routing strategy into PSO, allowing for generating a set of “optimal paths” to meet various requirements. In 2019, Yang Hui et al. proposed an efficient two-layer ant colony optimization algorithm for autonomous robot navigation [44]. An initial collision-free path is generated using a parallel elite ant colony optimization method. Subsequently, a path optimization algorithm, known as the turning point optimization algorithm, is applied to optimize the initial path in terms of length, smoothness, and safety, resulting in better collision-free paths indoors and outdoors. Nazarahari Milad et al. introduced a hybrid approach to path planning for multiple mobile robots in a continuous environment [45]. Firstly, an artificial potential field (APF) algorithm based on a deterministic scheme is proposed to find all feasible paths between start and end points in a discrete grid environment. Then, an Enhanced Genetic Algorithm (EGA) is developed using customized crossover and mutation operators to improve the initial paths in a continuous space and find optimal paths between start and end points. In 2020, Ajeil Fatin H. et al. proposed a hybrid PSO-MFB-based algorithm for solving the path planning problem of autonomous mobile robots in static and dynamic environments [46]. This algorithm consists of three modules: the optimized path formation using the PSO-MFB algorithm, infeasible point detection and conversion using a novel local search algorithm, and obstacle detection and avoidance to prevent collisions with obstacles. Experimental results demonstrate its efficiency and effectiveness in generating feasible paths even in complex dynamic environments. Liu Lijue et al. proposed an improved discrete bat algorithm to address premature convergence in the discrete bat algorithm (IDBA) [47]. This algorithm introduces a modified domain operator and utilizes the Floyd–Warshall algorithm to transform incomplete connectivity graphs into complete ones. Optimal paths satisfying constraints are then planned in the complete graph by simulating the foraging and obstacle avoidance process of bats. In 2021, Miao Changwei et al. proposed an Improved Adaptive Ant Colony Algorithm (IAACO) for robot path planning [48]. This algorithm introduces angle guidance and obstacle exclusion factors to accelerate real-time and safe path planning. Additionally, it incorporates heuristic information adaptive tuning and adaptive pheromone volatilization factors to balance convergence and global search capability. A multi-objective performance index is introduced to transform path planning into a multi-objective optimization problem, achieving comprehensive global optimization. Chen Jinchao et al. proposed an adaptive clustering algorithm based on symbiotic organisms searching for optimization strategies [49]. This algorithm efficiently generates feasible path schemes for each UAV, improving large-scale UAV swarm intelligence. In 2022, Deng Wu et al. proposed a multi-strategy particle swarm and ant colony hybrid optimization algorithm [50], MPSACO, to solve the path planning problem. This algorithm combines multi-strategy particle swarm optimization with a new pheromone initialization method for ACO, designing new pheromone allocation and updated strategies to enhance optimization performance. Chen Jinchao et al. proposed an ant colony system (ACS)-based algorithm to obtain paths that are good enough for UAVs efficiently [51]. Inspired by the foraging behavior of ants, this algorithm minimizes time consumption in cooperative search systems by finding near-optimal solutions.

In 2023, Huang Chen et al. proposed a novel and effective PSO algorithm [52], ACVDEPSO, to improve performance in various complex environments. This algorithm adaptively tunes parameters, cylinder vectors, and evolutionary operators, converting particle velocities to cylinder vectors to aid path search. Importantly, ACVDEPSO’s parameters are automatically selected based on the fitness values of time and particles. Additionally, a challenger based on the differential evolution operator is introduced to reduce the probability of local optima and speed up convergence. Lin Shiwei et al. drew inspiration from the SA algorithm to improve the PSO algorithm [53], proposing a hybrid PSO-SA algorithm for AGV path planning optimization. This algorithm balances global and local path exploration, planning high-quality paths while averaging average speeds. It reduces the average number of iterations by about 30% while maintaining path quality.

In 2024, Zhang Jie et al. proposed a collaborative path-planning method for heterogeneous AMVs for deep ocean exploration [54]. Integrating the Gray Wolf Optimizer (GWO) and Equalization Optimizer (EO), this method balances global and local path exploration, reducing local optima through conditional convergence factors. An adaptive surface path-planning method considers ocean currents and guides ASVs to collaborate in tracking underwater AUGs in the horizontal plane. Experimental results demonstrate the method’s advantages in underwater path planning for complex ocean exploration. Zhang Runda et al. proposed a two-layer trajectory optimization method [55]. It includes an efficient path planning layer and a fast trajectory planning layer. A novel Target Area Adaptive Rapid Exploration Random Tree Algorithm (TAA-RRT*) searches for the shortest path in the first layer. Then, a sampling target area is constructed based on the initial path for non-uniform sampling. The improved adaptive RRT* algorithm is used for sampling planning in the target area, combined with a direct connection strategy to locate the optimal solution quickly.

These studies have provided valuable insights into path planning algorithms, addressed various challenges, and provided multiple solutions to the 3D UAV trajectory and robot path planning problems. In short, the path planning problem has been popular among researchers. In this paper, we propose a novel meta-heuristic algorithm for path planning based on the above studies. The algorithm is used to solve the multi-UAV path planning problem in complex obstacle environments and considers multiple costs for optimization to plan comprehensive and high-quality paths for UAVs.

## 3. The Graduate Student Evolutionary Algorithm (GSEA)

In this section, we provide a detailed description of GSEA. Firstly, we delineate the source of inspiration for the algorithm. Next, we elucidate the process of initializing the algorithm, searching for a research direction, and concentrating on the research. Finally, we conduct a time complexity analysis.

### 3.1. Inspiration

Graduate students typically pursue further education after completing their undergraduate studies. In many countries, postgraduate education offers students advanced academic and professional training, facilitating in-depth research and specialized study in particular fields. Through postgraduate education, students broaden their knowledge and skill sets, enhancing their competitiveness and laying a solid foundation for future career development. In China, graduate students constitute approximately 10% of the total college population. The Chinese government actively promotes graduate education, implementing various measures to expand and enhance graduate enrollment to nurture high-level and innovative talents.

As graduate students, we begin our postgraduate journey by seeking supervisors aligned with our interests. Collaborating with a supervisor in our preferred direction allows us to delve into our chosen field. Upon entering the supervisor’s research group, we benefit from the guidance of accomplished peers who share our interests, fostering mutual progress. Additionally, we enrich our postgraduate life with personal pursuits such as relationships and travel, adding flavor to our academic endeavors. However, it is paramount to have a clear plan for our master’s degree journey, as it shapes the trajectory of our graduate student life. Whether to pursue further studies or enter the workforce becomes a pivotal decision for most master’s degree students, guiding our future aspirations.

Inspired by these experiences, we introduce a novel meta-heuristic method, GSEA. In the subsequent section, we explore the mathematical models underpinning these processes.

### 3.2. Mathematical Modeling of GSEA

#### 3.2.1. Initialization Stage-Examination/Preservation

Given that most graduate students currently have limited knowledge about potential advisors during the recommendation or entrance exam process, and mentors similarly gain only superficial understanding of students through one or two conversations, we simulate this behavior during the population initialization phase of GSEA. Like most heuristic algorithms, GSEA also generates candidate solutions by randomly generating candidate solutions within the given constraints of the problem and in each of its subsequent generations, the candidate solutions are updated according to the subsequent update rules [56] where the candidate solutions of GSEA are represented by Equation (1).

(1) $$X=\left[\begin{array}{ccccc} x_{1,1} & \ldots & x_{1,j} & x_{1,dim-1} & x_{1,dim}\\ x_{2,1} & \ldots & x_{2,j} & \ldots & x_{2,dim}\\ \ldots & \ldots & x_{i,j} & \ldots & \ldots \\ \vdots & \vdots & \vdots & \vdots & \vdots \\ x_{n-1,1} & \ldots & x_{n-1,j} & \ldots & x_{n-1,dim}\\ x_{n,1} & \ldots & x_{n,j} & x_{n,dim-1} & x_{n,dim} \end{array}\right]$$

where n denotes the population size (i.e., the number of graduate students), dim denotes the dimension of the given problem, and i, j are the indexes of the represented particles. $x_{i,j}$ is computed by Equation (2).

(2) $$x_{i,j}=\left(ub-lb\right)\times Rand+lb$$

where ub and lb are the bounded spaces given by the problem, i.e., each denotes the upper and lower bounds bounded by the problem, and Rand denotes one random number from 0 to 1.

#### 3.2.2. Finding Research Directions

**Figure 2:**
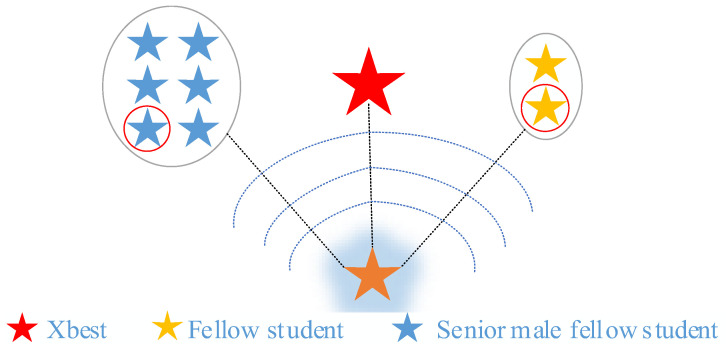
Graduate students searching for research directions are affected.

As an undergraduate, whether through a postgraduate guarantee or postgraduate entrance examination, he will generally make a two-way choice with his advisor after enrolling as a graduate student. After determining the instructor, the graduate students will study in the instructor’s group, study with the instructor and senior fellow students, and find their research direction, laying a good foundation for their subsequent development. However, mentors cannot entirely influence students’ behavior and research; often, senior peers and fellow students also exert significant influence. At the same time, fellow graduate students of the same class often greatly influence him. If his fellow students are outstanding, he will only be willing to live under others briefly. Inspired by the above behaviors, Equation (3) gives a model for graduate students searching for research directions.

(3) $$x_{i}^{new}=x_{i}+\left(r_{1}\times {Mentor}_{1}+r_{2}\times {Mentor}_{2}+P\times fellow\right)⊙randn$$

where $x_{i}^{new}$ denotes the graduate students after the guidance of teachers and elder brothers and sisters as well as the influence of fellow students, $x_{i}$ denotes the graduate students who have just started and have not yet begun to study, $r_{1}$, $r_{2}$ are the random numbers between 0 and 1, which are used to denote the acceptance degree of graduate students to the guidance of the teachers, elder brothers and sisters. randn is the normal distribution obeying the mean value of 0 and variance of 1, which is used to model the graduate students in the teachers, brothers and sisters and fellow students’ leadership, most of them are in the middle level, and a small number of them are in the excellent state. P is the degree of graduate students’ influence by fellow students, which is calculated by Equation (4). ${Mentor}_{1}$ is the mentor’s guidance to the graduate students, which is calculated by Equation (5); ${Mentor}_{2}$ is the guidance of the older siblings to the graduate students, which is calculated by Equation (6); and fellow is the inculcation of the graduate students by their fellow students, which is calculated by Equation (7).

(4) $$P=1-r_{1}-r_{2}$$

(5) $${Mentor}_{1}=x_{best}-x_{i}$$

where $x_{best}$ denotes the current optimal solution in the population and is used to model the graduate student’s advisor.

(6) $${Mentor}_{2}={Mentors}_{k1}-x_{i}$$

where Mentors contains the top three best solutions other than the optimal solution, the mean solution, the median solution, and the solution at the n−5 position, which is used to simulate a graduate student’s mentor. Since Mentors contains a total of 6 solutions, the value of k1 is a random integer from 1 to 6, which is used to simulate that we obtain a random guidance from an elder sibling while studying.

(7) $$fellow=x_{T1}-x_{i}$$

where T1 is a random integer from 1 to n used to simulate a fellow graduate student, $x_{T1}$ representing a random individual. Figure 2 illustrates the affected behaviors of graduate students in the process of finding a research direction.

In this subsection, the position update is affected by the optimal solution and the randomized mean solution, through which our introduction of normally distributed random numbers further guides the position update so that the position can better explore the global optimal solution without easily falling into the local optimal solution. By updating the above equation, the algorithm can work well in the exploration phase and achieve better results.

#### 3.2.3. Study Intensively

As a graduate student, after finding a research direction and obtaining guidance from his brothers and sisters, he must study according to his plans and work toward his dream life. Through reviewing relevant information and the general situation of the research group, we know that the proportion of graduate students who continue to study in the doctoral program is about 20%, so in GSEA, the population is divided into two “working” populations and “doctoral” population, of which the numbers of “working” and “doctoral” populations are represented by Equations (8) and (9), respectively.

(8) $$n_{1}=0.8*n$$

(9) $$n_{2}=0.2*n$$

Aristotle once said that man is a social animal. Graduate students are no exception, so socialization also affects their research. Here, this paper simulates the impact of socialization on graduate students, divided into two cases: those who are in love and those who are not. As a graduate student who is in love with a clear plan for the future and the companionship of a loved one, his or her life in the future will spiral upward with his or her efforts, so this paper uses the spiraling function to simulate this scenario; and as a graduate student who is not in love, although they may have a clear plan for the future, lacking the companionship of a loved one, there will always be some problems. As a graduate student who is not in love, although they may have a clear plan for the future, without the company of their lover, there will always be some “bumping around”, so we use the Levy flight function to simulate this scenario. At this stage, all graduate students were divided into two populations based on their plans for the future and updated individually. The update of graduate students in the “work” population is modelled by Equation (10). In Equation (10), the position update is guided by multiple positions, and the position update formula is selected by a randomization factor, which gives the algorithm a better randomization in the exploitation phase. In the process of position updating, we use spiral updating and Lévy flight respectively to make the algorithm converge faster and approach the global optimal solution as soon as possible, so that the algorithm obtains very good results.

(10) $$x_{i}^{new}=\left\{\begin{array}{c} x_{i}+({(Mentor}_{1}+{Mentor}_{2}+fellow+worker+lover)/5)*Spiral,\;rand<rand\\ x_{i}+({(Mentor}_{1}+{Mentor}_{2}+fellow+worker)/4)⊙Levy(dim)*M,\;else \end{array}\right.$$

where, worker denotes a senior who is ready to work after graduation, a randomly selected senior student from $n_{1}$. lover denotes one’s loved one, a randomly selected senior student from $n_{2}$. Spiral denotes a spiral flight function, the details of which are shown by Equation (11). M denotes an adaptive parameter, which is computed by Equation (12).

(11) $$Spiral=e^{Z*L}*\cos2\pi *L$$

(12) $$M=1+\frac{t}{T_{max}}$$

where L is calculated by Equations (13) and Z is calculated by Equation (14).

(13) L=2∗rand−1

(14) $$Z=e^{\cos\left(\pi *\left(1-\frac{t}{T_{max}}\right)\right)}$$

where t denotes the current iteration number and $T_{max}$ denotes the maximum iteration number. rand denotes a random number from 0 to 1. Levy(dim) denotes the Levy flight function. The calculation is performed by Equation (15).

(15) $$Levy=\frac{u}{{\left|v\right|}^{\frac{1}{\beta }}}$$

where β is the constant 1.5. u is calculated by Equation (16) and v is calculated by Equation (17).

(16) $$u=randn\left(1,\;dim\right)*{\left(\frac{\Gamma \left(1+\beta \right)\sin\left(\frac{\pi \beta }{2}\right)}{\Gamma \left(\frac{1+\beta }{2}\right)\beta 2^{\frac{\beta -1}{2}}}\right)}^{\frac{1}{\beta }}$$

(17) $$v=randn\left(1,dim\right)$$

Graduate student renewal for those who are prepared to continue their studies in the “doctoral” population after graduation is modeled by Equation (18).

(18) $$x_{i}^{new}=\left\{\begin{array}{c} x_{i}+({(Mentor}_{1}+{Mentor}_{2}+fellow+Phder+lover)/5)*Spiral,\;rand<rand\\ x_{i}+({(Mentor}_{1}+{Mentor}_{2}+fellow+worker)/4)⊙Levy(dim)*M,\;else \end{array}\right.$$

**Figure 3:**
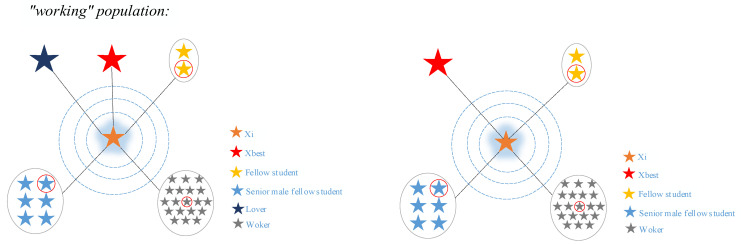
Schematic diagram of the guidance given to working population in the concentration phase of research.

**Figure 4:**
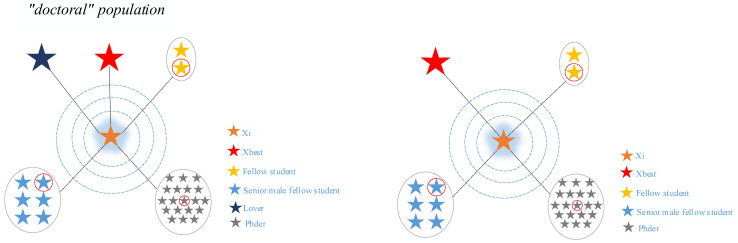
Schematic diagram of the guidance given to doctoral population in the concentration phase of research.

In this case, Phder denotes seniors who are preparing for doctoral studies after graduation. Figure 3 and Figure 4 show the overall mentoring received by these two groups of graduate students while concentrating on their research, respectively.

#### 3.2.4. Knowledge Accumulation

Graduate students progress through the above stages of study, influenced by their advisors and peers, accumulating knowledge along the way. This paper uses Equation (19) to model this phenomenon.

(19) $$x_{i}^{new}=\left\{\begin{array}{c} x_{i},\;\;{fitness}_{i}^{new}>{fitness}_{i}\\ x_{i}^{new},\;\;{fitness}_{i}^{new}<{fitness}_{i} \end{array}\right.$$

where ${fitness}_{i}$ denotes the fitness value of the original graduate student and ${fitness}_{i}^{new}$ denotes the fitness value of the mentored graduate student.

**Figure 5:**
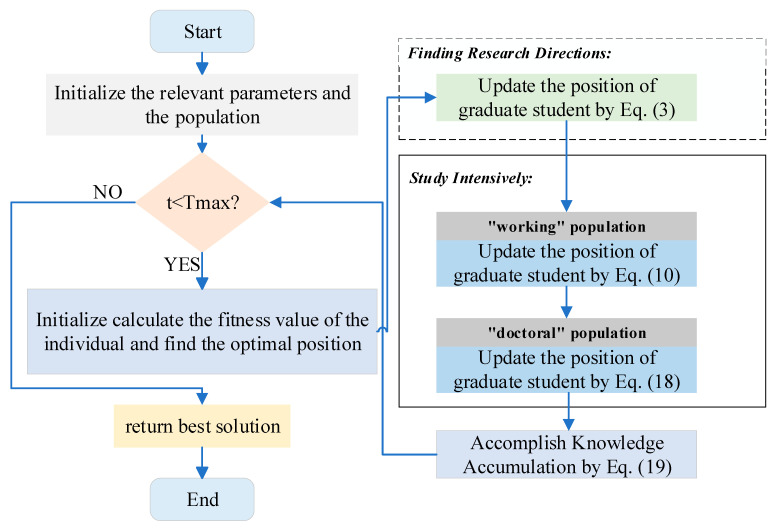
The flowchart of GSEA algorithm.

In summary, in GSEA, the optimization process starts with initialization to generate a set of random candidate solutions. Then, it goes through the search for research direction, concentration, and knowledge accumulation phases to find the optimal solution for the problem to be optimized. To visualize the understanding of this algorithm, Figure 5 shows the flowchart of the algorithm. Algorithm 1 gives the pseudo-code of the algorithm.
**Algorithm 1**: the pseudo-code of the GSEA1: Begin 2: Initialize the relevant parameters $3:\;\mathrm{while}\;t<T_{max}$
4:      Calculate the fitness of each graduate student 5:   Update the best solution 6:      Finding Research Directions: 7:          Update the position of graduate student by Equation (3) 8:      Study Intensively: 9:          “working” population: 10:          Update the position of graduate student by Equation (10) 11:        “doctoral” population: 12:          Update the position of graduate student by Equation (18) $13:\;\;\;\;\mathrm{Update}\;\mathrm{the}\;x_{i}$ and accomplish Knowledge Accumulation by Equation (19) 14:  End while 15:  return best solution 16: end

### 3.3. Algorithm Convergence Analysis

In intelligent optimization algorithms, whether an algorithm converges is a key factor in evaluating its performance. In this subsection, we analyze the convergence of GSEA based on Markov chains. First, we establish a Markov model for GSEA, then prove the algorithm’s convergence based on this model. The detailed steps are as follows:

#### 3.3.1. GSEA’s Markov Model

A Markov model represents a physical future state that depends solely on the current state, independent of past states, serving as a mathematical model for describing stochastic processes. The evolutionary processes of algorithms such as PSO and GWO can both be described as Markov processes. In GSEA, the position updates of individual graduate students depend solely on the current state, independent of past states, exhibiting no memory effect. This process is inherently stochastic. Therefore, this section employs a Markov model to represent this stochastic process. The following provides a detailed mathematical description and definition.

**Definition** **1.** *Graduate student individuals and graduate student individual state space. The state of a graduate student is defined by its position, denoted as* X*, where* X∈A*,* A *represents the feasible solution space. The set of all possible states for a graduate student constitutes its state space, denoted as* Y={X∣X∈A}.

**Definition** **2.** *Graduate Student Individual Population State and Graduate Student Individual Population State Space. The graduate student individual population state comprises the states of all graduate student individuals, denoted as*$\;s=\left(X_{1},X_{2},\ldots ,X_{N})\right.$*, where*$\;X_{i}\;$*represents the state of the* i*-th graduate student individual and* N *denotes the population size. The graduate student individual population state space consists of the set of all possible states of the graduate student individual population, denoted as*$\;S=\{s=(X_{1},X_{2},\ldots ,X_{N})|X_{i}\in Y,1⩽i⩽N\}$.

**Definition** **3.** *State equivalence. Define a function* ψ(s,X), ∀s∈S, X∈s*, denoted as*$\;\psi (s,X)=\sum_{i=1}^{N}\;X_{\left|X\right|}(X_{i})$*. Here,*$\;X_{|X|}\;$*denotes the characteristic function of an event;*$\;\psi \left(\begin{array}{c} s,X \end{array}\right)\;$*represents the number of postgraduate individuals in state* X *within the postgraduate population state* s*. If two postgraduate populations satisfy the condition*$\;s_{1},s_{2}\in S$*, for* ∀X∈Y*, if*$\;\psi \left(s_{1},X\right)=\psi \left(s_{2},X\right)$*, then*$\;s_{1}\;$*and*$\;s_{2}\;$*are considered equivalent, denoted as*$\;s_{1}∼s_{2}$.

**Definition** **4.** *State equivalence class. From equivalence relation*  “~”  *on*  S *, we can analogously derive the equivalence class of the individual postgraduate population state, denoted as*  Le=S/∼  *and referred to as the individual postgraduate population equivalence class. It possesses the following properties:*

**Property** **1.** *Within a given equivalence class* Le*, any individual graduate student population states are equivalent to one another, i.e.,*$\;s_{i}∼s_{j},\;\forall s_{i},s_{i}\in Le$.

**Property** **2.** *The state of any individual postgraduate population within* Le *is not equivalent to the state of any individual postgraduate population outside* Le*, denoted as*$\;s_{i}<\neq >s_{j},\;\forall s_{i}\in Le,\;\forall s_{j}\in Le$.

**Property** **3.** *Any two equivalence classes have no intersection, that is,* $\;Le_{1}\cap Le_{2}=\emptyset ,\;\forall Le_{1}\neq Le_{2}.$

**Definition** **5.** *Graduate Student Status Transition. For*$\;\forall X_{i}\in s,\;\forall X_{j}\in s$*, during the iteration of GSEA, the individual status of the graduate student transitions from*$\;X_{i}\;$*to*$\;X_{j}\;$*in one step, denoted as*$\;T_{s}(X_{i})=X_{j}$.

**Theorem** **1.** *In GSEA, the transition probability* $\;P(state\;X_{i}\;\to \;state\;X_{j})\;$ *for individual graduate students is expressed as*

(20) $$P\left(T_{s}\left(X_{i}\right)=X_{j}\right)=\left\{\begin{array}{l} P_{P1}\left(T_{s}\left(X_{i}\right)=X_{j}\right),\;\mathrm{Finding}\;\mathrm{research}\;\mathrm{directions}\;\mathrm{stage}\\ P_{P2}\left(T_{s}\left(X_{i}\right)=X_{j}\right),\;\mathrm{Study}\;\mathrm{intensively}\;\mathrm{stage} \end{array}\right.,$$

**Proof.**  Considering the individual postgraduate population as a set of points in hyperspace, the process of updating each postgraduate’s position represents the mutual transformation between sets of points within hyperspace. Based on Definition 3 and the geometric properties of GSEA, the transition probability for an individual postgraduate to move from state $X_{i}$ to state $X_{j}$ during the Finding research directions stage is:

(21) $$\begin{array}{c} P_{p_{1}}\left(T_{s}\left(X_{i}\right)=X_{j}\right)\\ =\left\{\begin{array}{cc} \frac{1}{|\left(\omega -I\cdot r-1\right)X_{i}+r\cdot X_{k}|}p_{1}\left(x_{i}\to x_{j}\right) & X_{j}\in \left[X_{i},\omega X_{i}+r\left(X_{k}-I\cdot X_{i}\right)\right]\;and\;F_{p_{i}}<F\\ \frac{1}{|\left(\omega +r-1\right)X_{i}-r\cdot X_{k}|}p_{1}\left(x_{i}\to x_{j}\right) & X_{j}\in \left[X_{i},\omega X_{i}+r\left(X_{i}-X_{k}\right)\right]\;and\;F_{p_{i}}\geq F_{i}\\ 0 & other \end{array}\right., \end{array}$$

where, $p_{1}\left(x_{i}\to x_{j}\right)$ can be expressed by Equation (22).

(22) $$p_{1}\left(x_{i}\to x_{j}\right)=\left\{\begin{array}{c} 1,\;\;\;\;\;f(x_{j})<f(x_{i})\\ 0,\;\;\;\;\;f(x_{j})⩾f(x_{i}) \end{array}\right.,$$

After a graduate student gains new insights during the Finding Research Directions stage, they must proceed to the Study Intensively stage to explore whether better solutions exist. Therefore, the transition probability from state 1 to state 2 during the Study Intensively stage is

(23) $$P_{p_{2}}\left(T_{s}\left(X_{i}\right)=X_{j}\right)=\left\{\begin{array}{c} \frac{1}{|\left(\omega +p\cdot r\left(ub-lb\right)-1\right)X_{i}+\rho \cdot lb|}p_{2}\left(x_{i}\to x_{j}\right)\\ X_{j}\in \left[X_{i},\omega X_{i}+\rho \cdot \left(lb+r\left(ub-lb\right)X_{i}\right)\right]\\ 0,\;\;\mathrm{other} \end{array}\right.,$$

where $p_{1}\left(x_{i}\to x_{j}\right)$ can be expressed by Equation (24).

(24) $$p_{2}\left(x_{i}\to x_{j}\right)=\left\{\begin{array}{c} 1,\;\;\;\;\;f\left(x_{j}\right)<f\left(x_{i}\right)\\ 0,\;\;\;\;\;f\left(x_{j}\right)⩾f\left(x_{i}\right) \end{array}\right.,$$

where X is multidimensional, the volume of the hyperspace cube is represented by its absolute value, and vector addition/subtraction is denoted by the plus/minus sign. Since GSEA is implemented through two stages, the transition probability of a graduate student’s status shifting from state $x_{i}$ to state $x_{j}$ is jointly determined by Equations (21)–(24) upon completion of certification. □

**Definition** **6.** *Probability of state transition for individual graduate student populations. For state* $\forall s_{i}\in S,\forall s_{j}\in S$*, during the GSEA iteration process, the individual graduate student population state transitions from* $s_{i}$ *to* $s_{j}$ *in one step, denoted as* $T_{S}(s_{i})=s_{j}$*. The transition probability for an individual graduate student population state to shift from* $s_{i}$ *to* $s_{j}$ *in one step can be expressed as:*

(25) $$P\left(T_{S}\left(s_{i}\right)=s_{j}\right)=\prod_{N}^{m=1}\;P\left(T_{s}\left(X_{im}\right)=X_{jm}\right),$$

The probability of a graduate student individual transitioning from state $s_{i}$ to state $s_{j}$ is the probability that all graduate student individuals in population $s_{j}$ simultaneously transition to the state of all graduate student individuals in population $s_{j}$.

**Theorem** **2.** *In GSEA, the individual population state sequence* {s(t):t>0} *for graduate students is a finite Markov chain.*

**Proof.**  The search space in any optimization algorithm is finite, hence the number of states $X_{i}$ in any individual graduate student is finite. Therefore, the state space Y of an individual graduate student is finite. The state space $s=(X_{1},X_{2},\ldots ,X_{N})$ of a graduate student population consists of N graduate students, where N is a finite positive integer. Thus, the state space S of the graduate student population is finite. □

From the state transition probabilities of the individual postgraduate population, it follows that in the state sequence {s(t):t>0} of the individual postgraduate population, for any s(t)∈S,s(t+1)∈S, their transition probability $P\left(T_{S}(s(t))=s(t+1))\right)$ is determined by the state transition probabilities $P(T_{s}(X(t)=X(t+1))$ of all postgraduate individuals within the population. By Theorem 1, the state transition probability $P(T_{s}(X(t)=X(t+1))$ for any individual in the graduate student population depends solely on the state X(t) at time t, the random factor r, and the upper and lower bounds of the search space. Therefore, $P(T_{s}(s(t))=s(t+1))$ is also solely dependent on the state at time t, meaning that the state sequence {s(t):t>0} of the graduate student population exhibits Markov property. Furthermore, since the state space is countable, the state space S of the graduate student population is finite. Consequently, it forms a finite Markov chain.

By Theorem 1, $P(T_{s}(X(t)=X(t+1))$ depends only on the state X(t) at time t, and not on the time t itself. Therefore, the individual population state sequence {s(t):t>0} of graduate students is a finite homogeneous Markov chain.

#### 3.3.2. GSEA Convergence Analysis

**Definition** **7.** *Optimal State Set* G. *For an optimization problem* ⟨Y,f⟩ *where the global optimum solution is* $g_{b}$ *and the optimal state set is defined as* $G=\{s^{*}=(X_{1},X_{2},\ldots ,X_{N})|f(X)=f(g_{b}),s\in S\}$, *we consider the case of* G⊂S. *If* G=S, *then the solution set within the feasible solution space would also be the optimum solution, rendering the optimization meaningless.*

**Definition** **8.** *Optimal Population State Set* H*. For the global optimum solution of an optimization problem* ⟨Y,f⟩*, the optimal population state set is defined as* $H=\{q=(s_{1},s_{2},\ldots ,s_{n})|\exists s_{i}\in G,1⩽i⩽n\}$.

**Lemma** **1.** *Suppose a Markov chain has a nonempty closed set* E*, and there exists no other nonempty closed set* O *such that* E∩O=∅ *holds. Then when* j∈E *holds,* $\underset{n\to \infty }{lim}\;P(X_{n}=j)=\pi_{j},$ *holds, and when* j∉E *holds,* $\underset{n\to \infty }{lim}\;P(X_{n}=j)=0$ *exists.*

**Theorem** **3.** *As the number of iterations approaches infinity, the sequence of population states converges to the optimal population state set* H.

**Proof.**  In GSEA, the individual position update strategy employs a best-individual retention mechanism. As iterations progress, better values than previous ones are obtained; when a worse value than the previous one appears, no update occurs. Therefore, $P(X_{k+1}\notin G|X_{k}\in G)=0$, and G is a closed set with probability 1. □

By Definition 8, $\exists s_{i}\in G$, the Kth iteration has $q_{k}=(s_{1},s_{2},\ldots ,s_{n})\in H$. In a closed set G, there must exist $X_{k+1}\in G$. At iteration k+1, the population state is $q_{k+1}=(s_{1},s_{2},\ldots ,s_{n})\in H$. Therefore, *$P(q_{k+1}\notin H|q_{k}\in H)=0$*. It follows that H is a closed set in the state space S.

Suppose there exists a non-empty closed subset M in the state space S, and M∩H=∅. Let $q_{i}=(g_{b},g_{b},\ldots ,g_{b})\in H,\forall q_{j}=(s_{j1},s_{j2},\ldots ,s_{jn})\in M$, Existence $f(s_{jc})>f(g_{b})$. From the Chapman–Kolmogorov equation, we obtain $P_{s_{i},s_{j}}^{l}=\sum_{s_{r_{1}}\in ss_{n-1}\in s}\;P\left(T_{s}\right.(s_{i})=s_{n_{1}})P(T_{S}(s_{n_{1}})=s_{n_{2}})\cdots P(T_{S}(s_{n-1})=s_{j})$.

Therefore, for each transition probability $P(T_{s}(s_{r_{c+i}})=s_{r_{c+i+1}})$ in the C-K equation $P_{s_{i},s_{j}}^{l}$ during infinite iterations, Equation (20) holds, i.e., $P(T_{s}(s_{r_{c+i}})=s_{r_{c+i+1}})>0$. In summary, M is not a non-empty closed set, contradicting the assumption. Hence, in the state space S, there exists only one closed set H, which does not contain any closed sets other than G.

In summary, Theorem 3 follows directly from Lemma 1.

**Lemma** **2.** *Global Convergence Theorem. Suppose* f *is a measurable function, and* A *is a measurable subset of* $R^{n}$*. Then* f *satisfies the following conditions:*

*(a)*  $f\left(D\left(x,\zeta \right)\right)⩽f\left(x\right),$ *if* ζ∈A*, then* f(D(x,ζ))⩽f(ζ).
*(b)*  *For any Borel subset* B *of* A*,* s.t.v[B]>0*, then,* $\prod_{\infty }^{k=0}\;\left(1-u_{k}\left[B\right]\right)=0$*, and* $\{x_{k}\}\overset{\infty }{k=0}$ *is the sequence generated by algorithm, specifically* $\underset{k\to \infty }{lim}P(x_{k}\in R_{\varepsilon ,M})=1\;$*, where* $P\left(x_{k}\in R_{\varepsilon ,M}\right)$ *is the probability measure of the solution* $x_{k}$ *at step* k *of the algorithm.*

**Theorem** **4.** *GSEA exhibits global convergence. By Lemma 2, for GSEA, the current optimal solution in the iteration process is saved as*

(26) $$X_{i}\left(t\right)=\left\{\begin{array}{c} X_{i}^{new}\left(t+1\right),\;\;F_{i}^{new}<F_{i}\\ X_{i}\left(t\right),\;\;F_{i}^{new}⩾F_{i} \end{array}\right.,$$

Therefore, each iteration of GSEA preserves the optimal position within the population, clearly satisfying condition (a). This indicates that the current optimal solution is always retained during iteration. By Theorem 3, as the number of iterations approaches infinity, the population state sequence converges to the optimal population state set H. This indicates that after a sufficiently large number of iterations or after infinite iterations, the population state sequence converges to the optimal set. Therefore, the probability of failing to find the global optimum is zero, yielding $0<\mu_{k}[B]<1$. That is, $\prod_{\infty }^{k=0}\;\left(1-\mu_{k}\left[B\right]\right)=0$ satisfies condition (b). Thus, the global convergence theorem establishes that GSEA is a globally convergent algorithm.

### 3.4. Time Complexity Analysis

In some fields, not only is the algorithm required to have good performance, but also it is required to be able to plan our work in real time. So, evaluating the goodness of an algorithm needs to focus not only on the performance, but usually also on its time complexity. In this case, even if the algorithm has a good performance, not being able to plan the tasks in real time will make the algorithm look like a very cockamamie performance. Therefore, in this subsection, we analyze the overall time complexity of GSEA, whose complexity is mainly expressed by the population initialization and the main algorithmic functions. In terms of population initialization, since the number of populations we set is n, the time complexity of its initialization process is O(n); for the main function of the algorithm, it mainly consists of two parts: finding the research direction and concentrating on the research, in which we use T to denote the maximal number of iterations, and dim to denote the dimensionality of the problem, so the time complexity of this part is O(n∗T)+O(2× n∗T∗dim); in summary, the time complexity of GSEA proposed in this paper is O(n∗T∗(2×dim+1)).

## 4. Numerical Optimization Experiments

In this section, we evaluate the performance of the GSEA algorithm proposed in this paper by subjecting it to tests on different test sets. Firstly, we analyze the convergence behavior of the algorithm. Next, we conduct a quantitative analysis by comparing it with 11 other state-of-the-art algorithms across 42 test functions in two test sets, CEC2017 and CEC2022. Furthermore, to discern its variances from other competing algorithms, we conduct statistical analyses, including the Wilcoxon rank sum test and the Friedman mean rank test, to comprehensively and integratively assess the algorithm’s performance. All experiments in this section were conducted using the MATLAB 2023a platform.

### 4.1. Benchmark Test Functions

For evaluating the performance of an algorithm, the benchmarking function plays an extremely important role in providing an evaluation standard for our algorithms, so that we can clearly see how good and bad each algorithm is on the standard test set as well as its strengths and weaknesses. In this paper, we have utilized two test sets, CEC2017 [57] and CEC2022 [58], to evaluate our proposed algorithms. The details of 41 test functions in the two test sets CEC2017 and CEC2022 are listed in Table 1 and Table 2, respectively.

### 4.2. Competitor Algorithms and Parameters Setting

In this paper, we compare GSEA with 11 other state-of-the-art algorithms and 4 other human-based algorithms including Dung beetle optimizer (DBO), Red-tailed hawk algorithm (RTH), Rime optimization algorithm (RIME), artificial jellyfish searcher (JS), sparrow search algorithm (SSA), butterfly optimization algorithm (BOA), Horned Lizard Optimization Algorithm (HLOA), Human Evolutionary Optimization Algorithm (HEOA), CPSOGSA, HPHHO, Grey wolf optimizer based on Aquila exploration method (AGWO). Table 3 summarizes the parameter settings of these compared algorithms, where n denotes the population size and T denotes the maximum number of iterations, which we set to 30 and 1000 for a fair comparison with each of the competing algorithms.

### 4.3. Analysis of the Convergence Behavior

**Figure 6:**
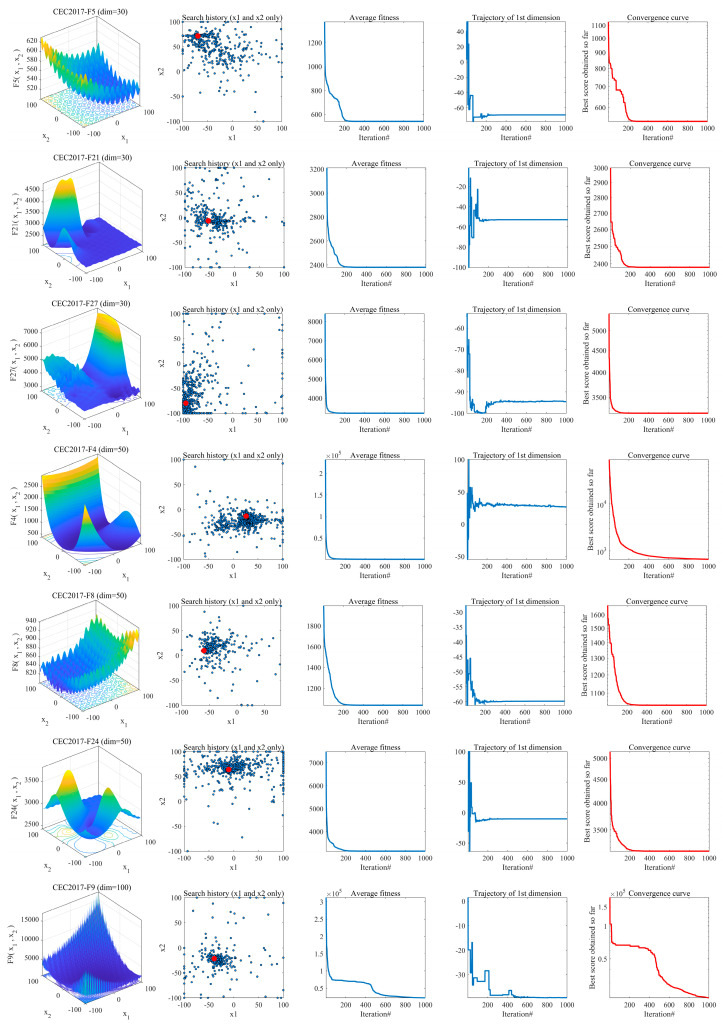

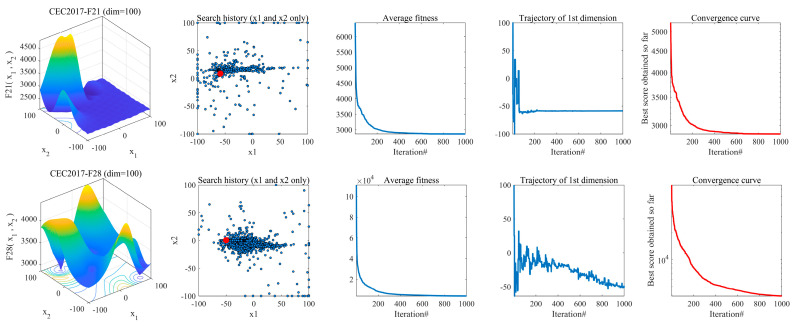
Convergence behavior of GSEA in the search process.

Analyzing the convergence of an algorithm is fundamental to evaluating its performance [70]. To assess the convergence of GSEA, we designed an experiment employing four metrics: search history, average fitness value, trajectory, and convergence curve. The experiment begins with 30 individuals randomly distributed in a two-dimensional search space and aims to find the solution within 1000 iterations. The search space of the test function is depicted in the first column of Figure 6. In the second column, the search history illustrates the movement trajectory of individuals throughout the evolutionary process, with small blue dots representing individual movements and red dots indicating the global optimal solution. The experimental results demonstrate that the GSEA algorithm effectively explores the search space and approximates the optimal solution.

The third column showcases the change in the average fitness value over iterations. Initially, the average fitness value is high, but it rapidly decreases as the algorithm progresses, indicating that most search particles gravitate towards or near the optimal solution location. The fourth column displays the search trajectories of particles, with the trajectory metric revealing fluctuations during exploration and gradual stabilization during exploitation. This indicates the algorithm’s adeptness at both exploration and exploitation.

The final column presents the convergence curve of the algorithm. The experimental results depict the algorithm’s continuous ability to escape local optimal solutions and eventually converge to the global optimal solution as iterations proceed. In summary, the analysis of experimental results indicates that this algorithm exhibits convergence behavior, validating its effectiveness.

### 4.4. Parameter Sensitivity Analysis

Since the Yuan heuristic algorithm heavily relies on parameter settings, these settings significantly impact the algorithm’s performance. Therefore, in this subsection, we conduct a parameter sensitivity analysis for GSEA, with detailed specifics as follows:

#### 4.4.1. Population Size Sensitivity Analysis

**Figure 7:**
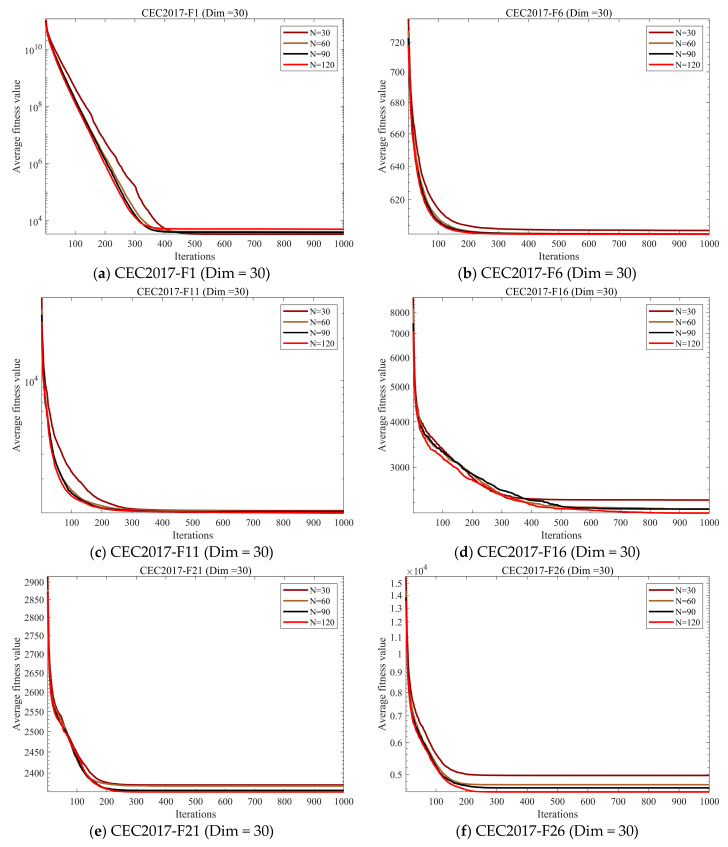

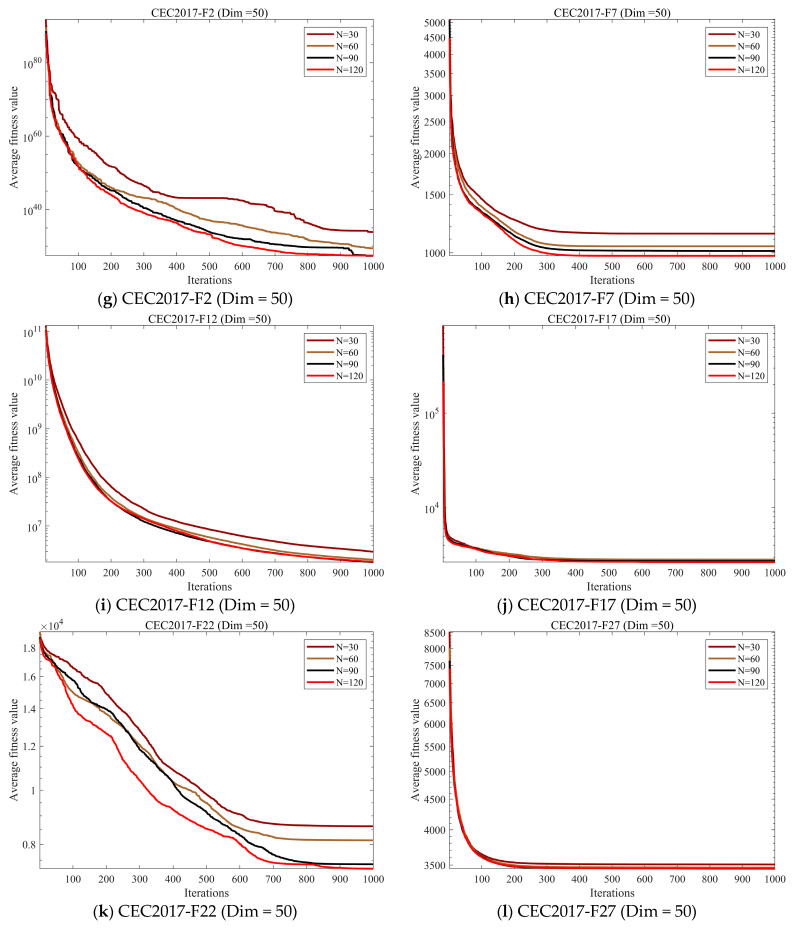

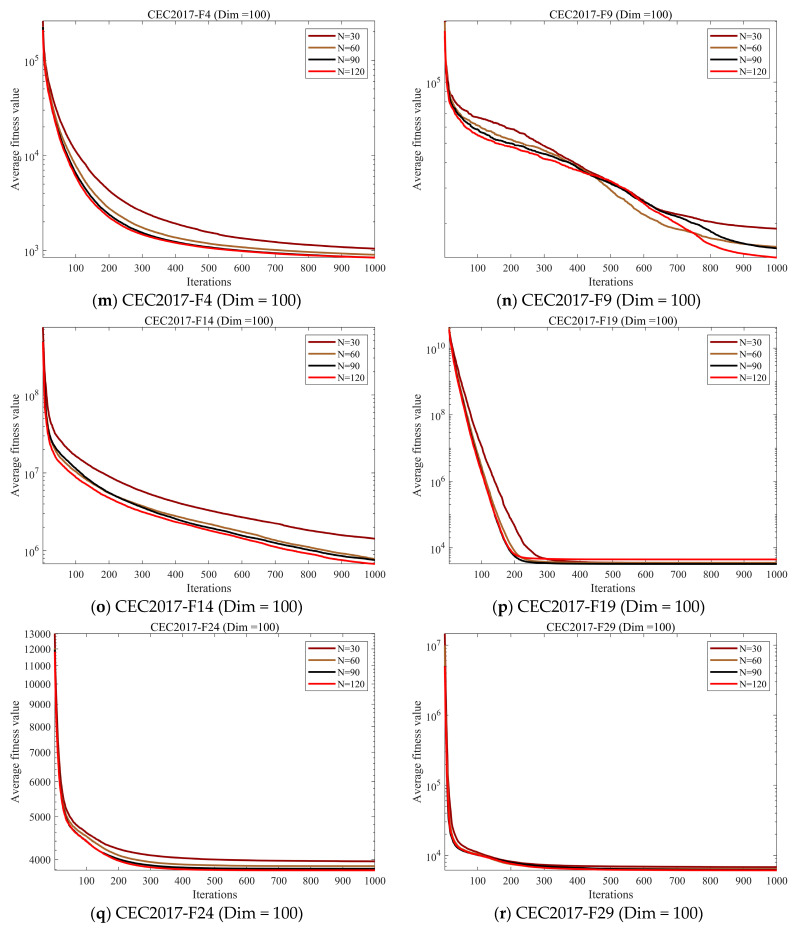
Population size sensitivity analysis convergence curve.

In this section, we conducted a sensitivity analysis to explore the impact of population size on algorithm performance. With the iteration count fixed at 1000, we set the population size to 30, 60, 90, and 120, respectively. Experimental analysis was performed across these four scenarios to investigate the convergence of GSEA. Figure 7 illustrates the convergence of GSEA across four scenarios.

The experimental results demonstrate that GSEA converges effectively regardless of whether the population size is set to 30, 60, 90, or 120. However, there are slight differences in convergence speed or accuracy across different population sizes. To ensure fairness in comparisons with other algorithms in subsequent experiments, we will uniformly set the GSEA population size to 30.

#### 4.4.2. Sensitivity Analysis of Iteration Count

**Figure 8:**
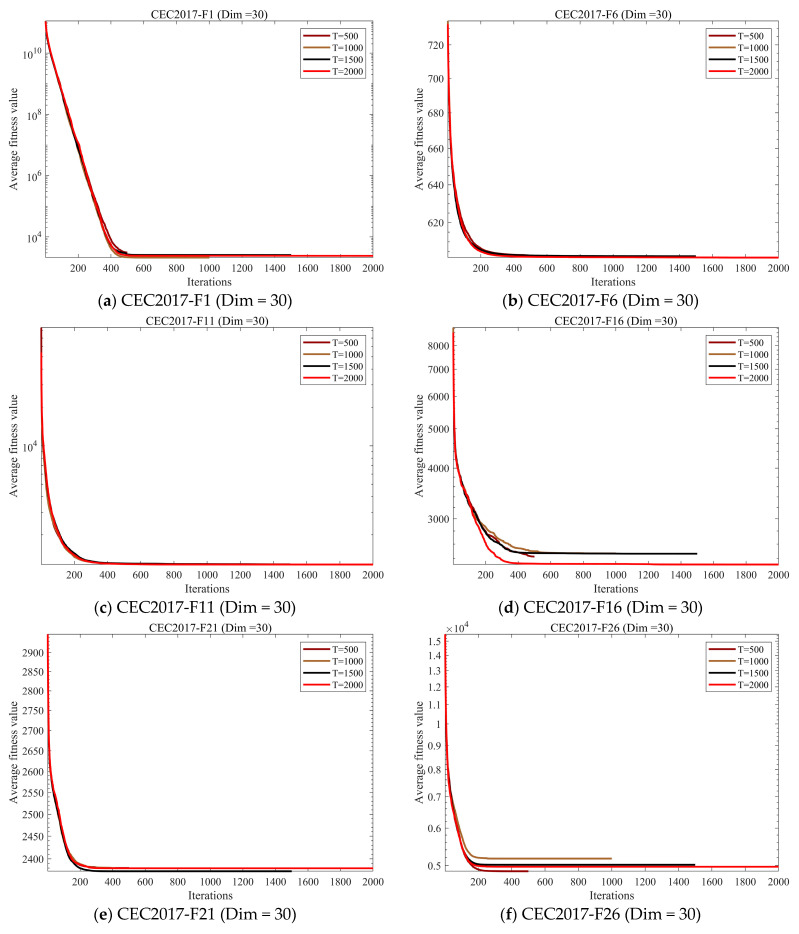

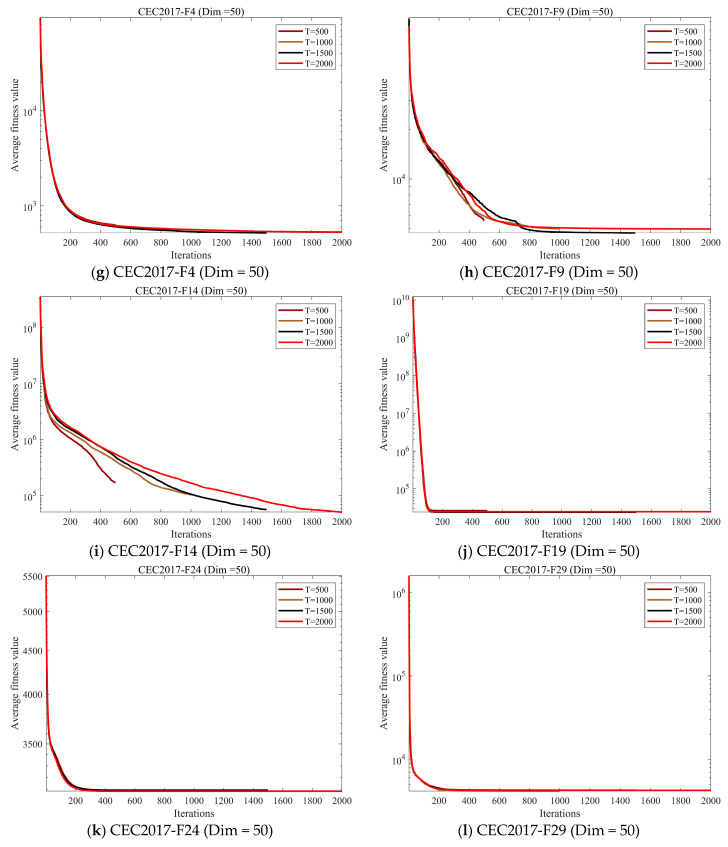

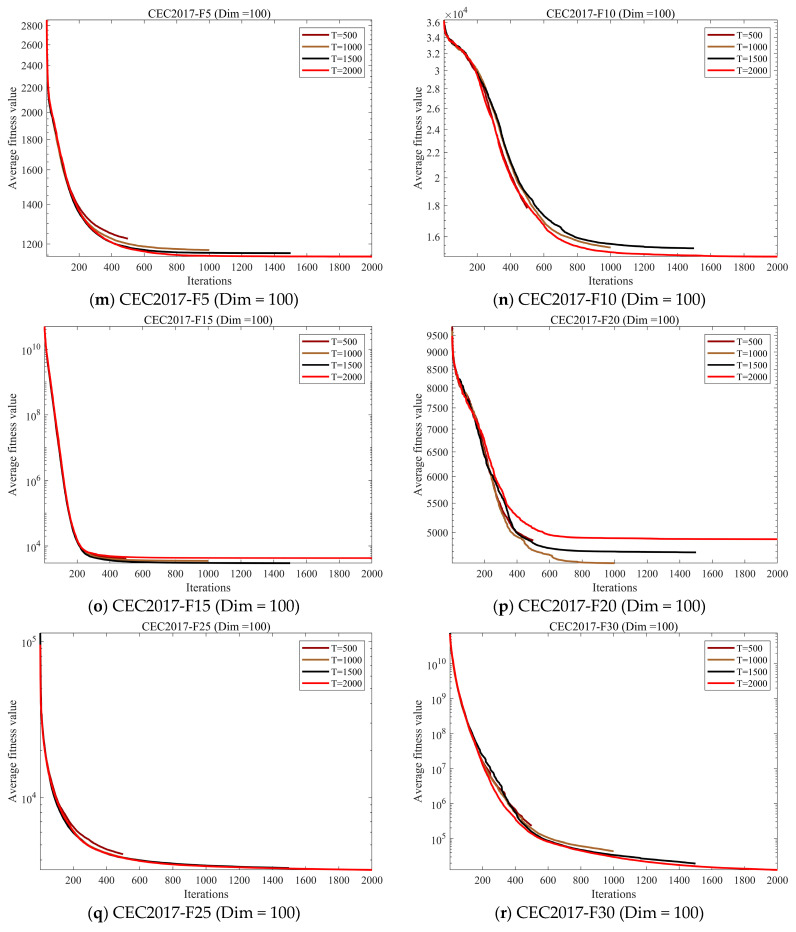
Sensitivity analysis of iteration count convergence curve.

In this section, to investigate the impact of iteration count on GSEA performance, we fixed the population size at 30 and conducted experimental analyses with iteration counts set to 500, 1000, 1500, and 2000. The experimental results are shown in Figure 8.

The experimental results indicate that when the iteration count is 500, GSEA does not converge for some test functions. However, as the iteration count increases, GSEA tends toward convergence. For the majority of test functions, GSEA converges when the iteration count reaches 1000. Therefore, to ensure a fair comparison with other algorithms in subsequent experiments, we set the iteration count for all algorithms to 1000.

### 4.5. Quantitative Evaluation

In this section, to evaluate the performance of the GSEA algorithm, we compare the performance of GSEA as well as the comparison algorithms on numerical optimization experiments using the CEC2017 and CEC2022 test sets.

#### 4.5.1. Compare Using CEC 2017 Test Functions

**Figure 9:**
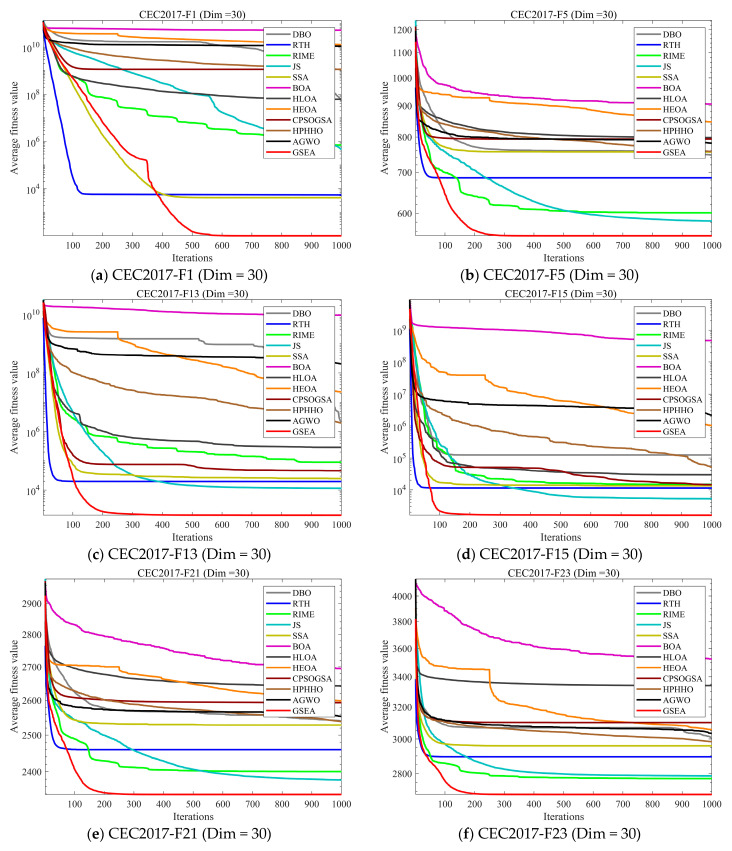

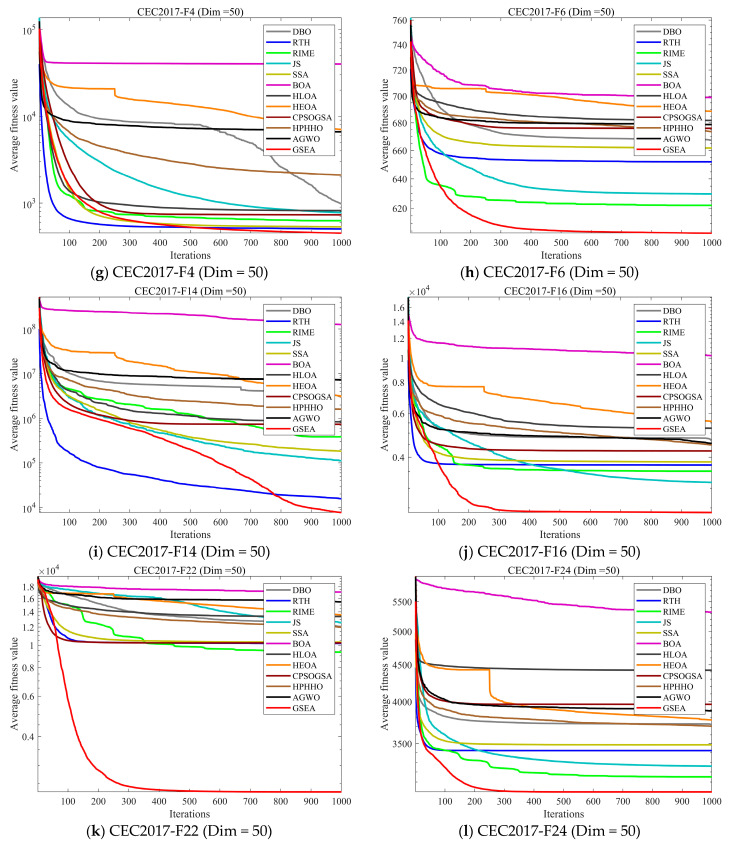

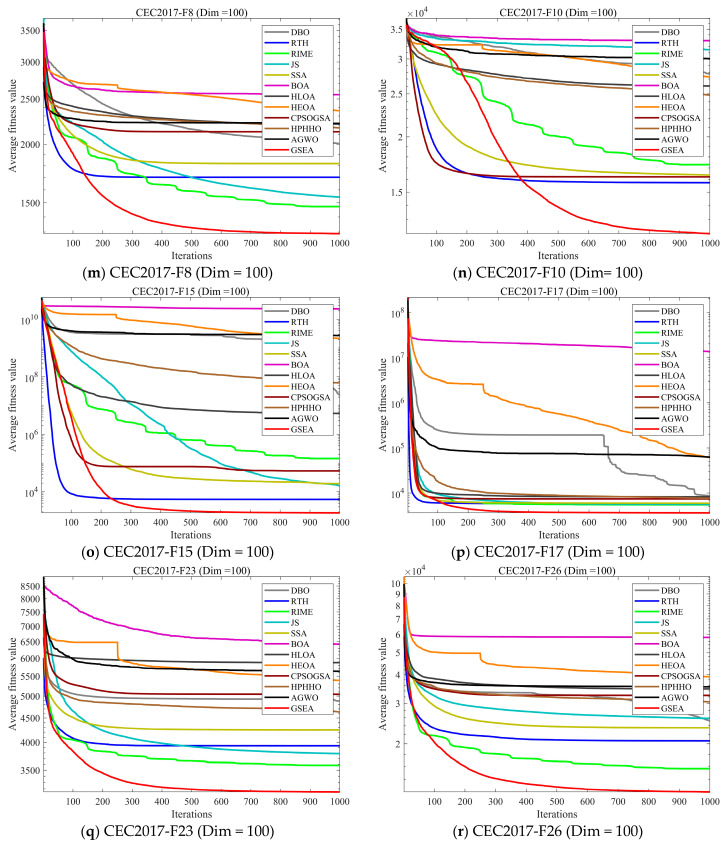
Comparison of convergence speed of the state-of-the-art algorithms on CEC2017 test set.

**Figure 10:**
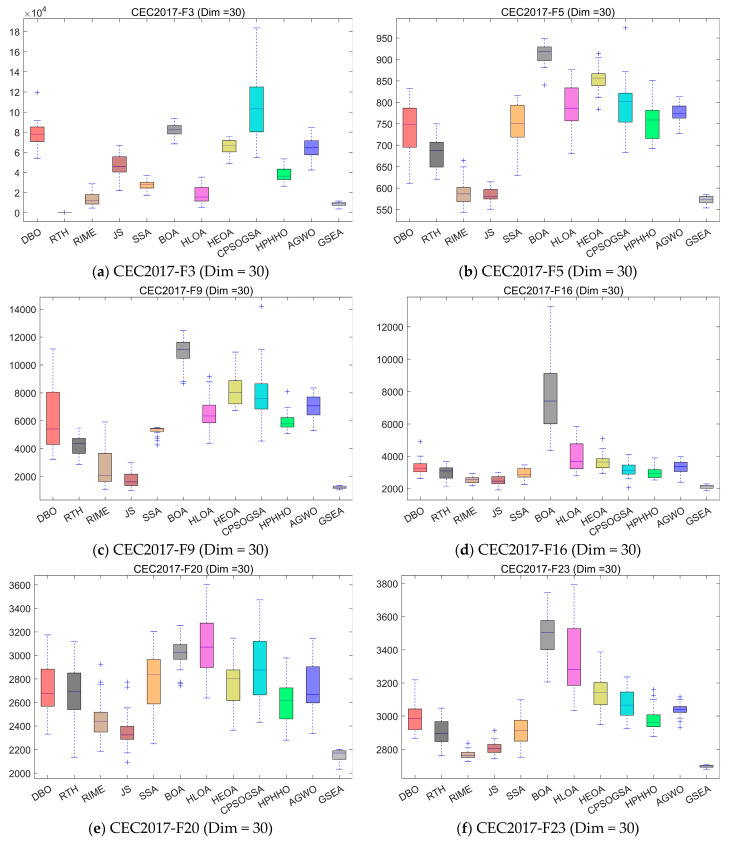

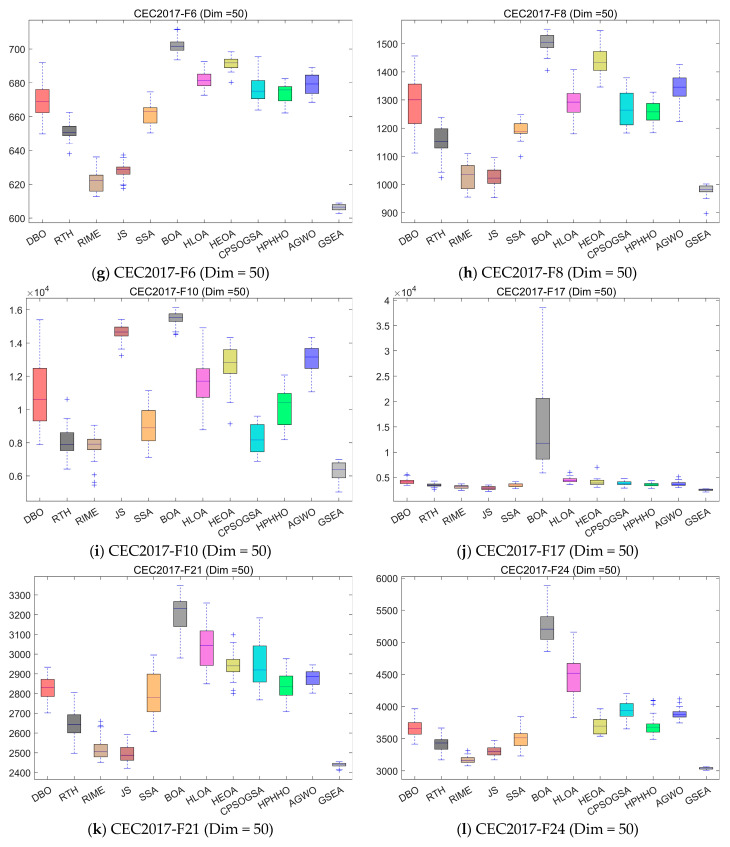

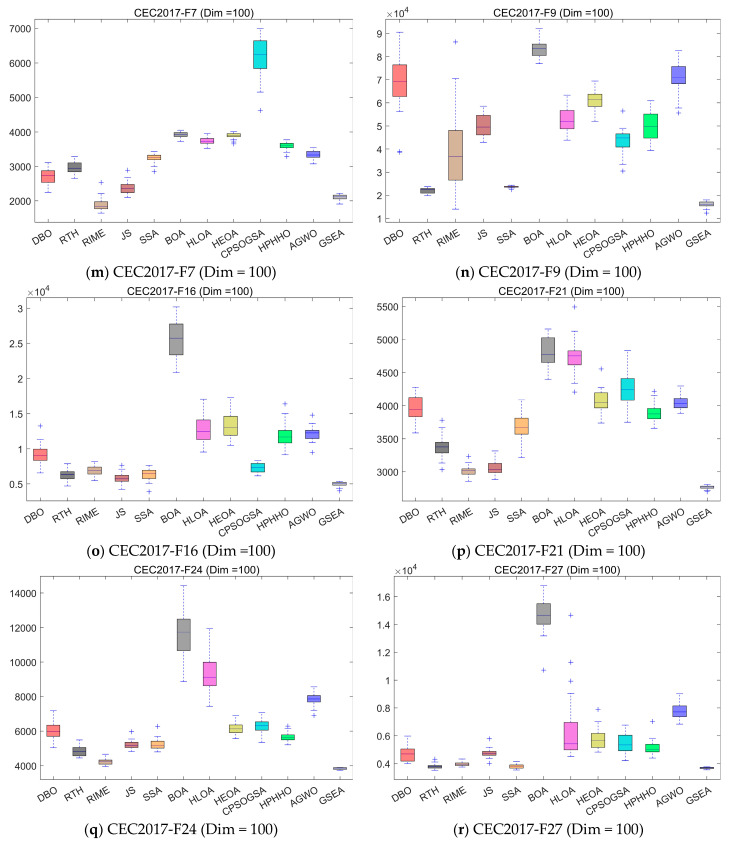
Boxplot analysis for the state-of-the-art algorithms on the CEC2017 test set.

In this subsection, we evaluate the performance of GSEA using the CEC2017 test set, which we evaluate on three dimensions: 30, 50, and 100 dimensions. Table 4, Table 5 and Table 6 present the mean and standard deviation of 30 independent runs of each algorithm on each test function in the three dimensions. Among them, Ave represents the mean of 30 runs, and std represents the standard deviation of 30 runs. To further demonstrate the performance of GSEA, Figure 9 shows the convergence curves of GSEA compared with 11 other advanced algorithms. Figure 10 shows the boxplots of 30 independent experiments, which comprehensively demonstrate the performance of GSEA.

As shown in Figure 9, GSEA outperforms the comparison algorithms on most test functions. However, it is inevitable that at F4 (dim = 50), GSEA converges slower than RTH. Although its final convergence accuracy is slightly better than RTH, the slow convergence speed remains a significant drawback of GSEA, indicating that its performance on unimodal functions can be further improved. Nevertheless, since the primary problem addressed in this paper is complex UAV path planning, GSEA demonstrates significant advantages over the comparison algorithms on mixed and composite functions.

Figure 10 presents box plots comparing GSEA with 11 other algorithms across three dimensions of CEC2017, based on 30 runs. From the figure, we can observe that although GSEA’s optimal values are not the best in some cases, it demonstrates the highest stability. For UAV path planning problems, ensuring the safety and effectiveness of mission execution is a critical metric. Therefore, the stability of path planning algorithms is highly valued. The test results clearly indicate that GSEA possesses this advantage.

#### 4.5.2. Compare Using CEC 2022 Test Functions

**Figure 11:**
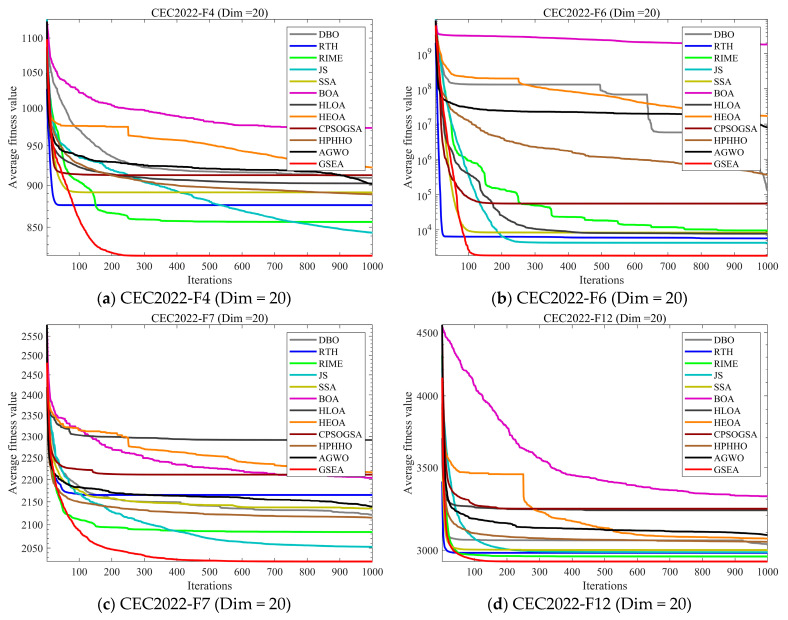
Comparison of convergence speed of the state-of-the-art algorithms on CEC2022 test set.

**Figure 12:**
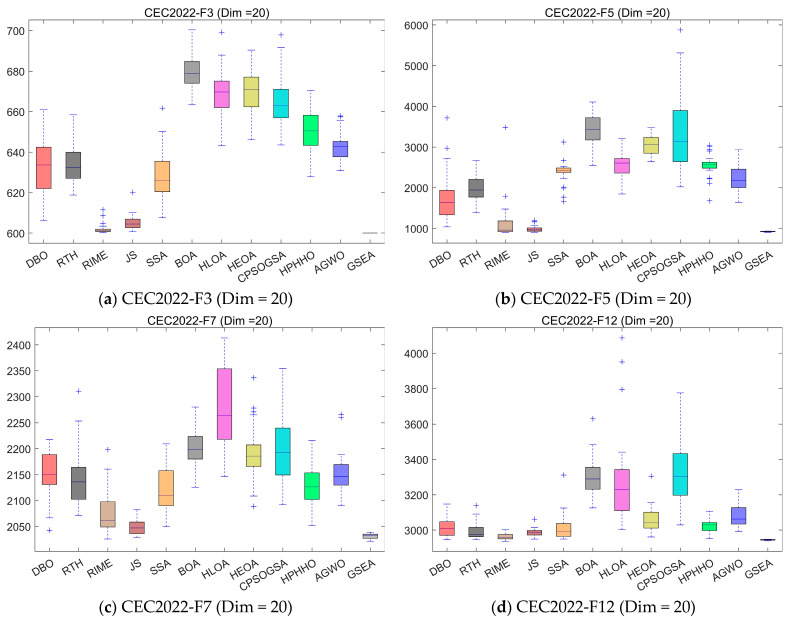
Boxplot analysis for the state-of-the-art algorithms on the CEC2022 test set.

In this subsection, in order to explore the performance of GSEA more comprehensively, we evaluate it using the CEC2022 test set and analyze it by comparing GSEA with 11 other state-of-the-art algorithms on 20 dimensions. Figure 11 shows the convergence curves of the comparison. Figure 12 shows the boxplots of 30 independent runs of the algorithms, for a comprehensive evaluation of the performance of the algorithms. Table 7 presents the mean and standard deviation of 30 independent runs of each algorithm on each test function in the three dimensions. Among them, Ave represents the mean of 30 runs, and std represents the standard deviation of 30 runs.

As shown in the experimental results of Figure 11, on the CEC2022 test set, the GSEA algorithm demonstrates significant advantages over other comparison algorithms in both convergence accuracy and convergence speed. It is particularly noteworthy that although GSEA converges more slowly than RTH for certain functions, its convergence accuracy significantly surpasses that of RTH. For the unmanned aerial vehicle path planning problem we aim to solve, sacrificing a portion of convergence speed to achieve substantially higher convergence accuracy is well worth it.

As shown in the experimental results of Figure 12, the proposed GSEA algorithm exhibits minimal fluctuation across 30 runs on various test functions, demonstrating its stable performance and establishing it as a highly promising path planning algorithm for unmanned aerial vehicles.

### 4.6. Statistical Analysis

In this section, we analyze the experimental results using the Wilcoxon rank sum test and the Friedman mean rank test to statistically examine the differences between GSEA and other comparison algorithms.

#### 4.6.1. Wilcoxon Rank Sum Test

In order to verify the performance of GSEA more comprehensively, in this subsection, we will use the Wilcoxon rank sum test to evaluate whether the results of this algorithm’s runs are significantly different from the other algorithms at the *p* = 0.05 level of significance [71]. We denote the original hypothesis as $H_{0}$, indicating that there is no significant difference between the two algorithms. If *p* < 0.05, we reject the original hypothesis indicating that there is a significant difference between the two algorithms. If *p* > 0.05, we accept the original hypothesis indicating that there is no significant difference between the two algorithms. Table 8, Table 9, Table 10 and Table 11 give the results of GSEA on the CEC2017 and CEC2022 test sets, respectively. The tabular data shows that GSEA achieved competitive results.

#### 4.6.2. Friedman Mean Rank Test

In this subsection, we utilize the CEC2017 and CEC2022 test sets to conduct the nonparametric Friedman mean rank test [72], aiming to evaluate the experimental results of GSEA and compare them with other comparison algorithms on the test sets.

Table 12 presents the Friedman mean rank of 12 algorithms across various dimensions on two test sets, where 11 denotes the average rank across 30 test functions and 22 indicates the final rank across 30 test functions. The experimental results demonstrate that the proposed GSEA achieved first place in all four scenarios, securing a significant advantage. In particular, GSEA achieved the top rank on numerous functions, resulting in an average ranking no greater than 2 across all tested functions. In contrast, each of the competing algorithms had an average ranking exceeding 2.

## 5. 3D UAV/Robot Path Planning

In our previous experiments, we evaluated the performance of GSEA by benchmarking it against standard test functions and conducted statistical analyses to compare its performance with other algorithms. However, a robust algorithm should not be limited to test set evaluations; it should also demonstrate efficacy in real-world applications.

In this section, we apply the GSEA algorithm to real-world problems, starting with 3D UAV path planning. We compare its performance with other popular algorithms to determine whether GSEA outperforms them in real-world scenarios. Furthermore, to assess the algorithm’s robustness, we apply it to the robot path-planning problem. We randomly generate maps and task the algorithm with path planning for the robot across various scenarios. Through these experiments, we aim to ascertain whether the algorithm can consistently generate high-quality paths for the robot across diverse situations.

### 5.1. 3D UAV Path Planning

In recent years, propelled by ongoing technological advancements, unmanned aerial vehicles (UAVs) have found widespread applications across various industries. Path planning is pivotal in ensuring that UAVs efficiently execute tasks assigned by technicians. Consequently, UAV path planning has emerged as a prominent research focus in contemporary times [73].

To address the UAV path-planning problem, this subsection endeavors to simulate real-world scenarios and establish a comprehensive model.

#### 5.1.1. Modeling of the UAV Flight Environment

One of the most critical tasks in planning UAV paths is to model the UAV flight environment. We use a composite function to model the UAV flight environment in this model. The baseline terrain is shown in Equation (27).

(27) $$z_{1}\left(x,y\right)=2\sqrt{2}\cos\left(\frac{x-y}{2}\right)\cos\left(\frac{\pi }{4}-\frac{x+y}{2}\right)+\sin\left(\sqrt{x^{2}+y^{2}}\right)+\cos\left(\sqrt{x^{2}+y^{2}}\right)$$

where, since $z_{1}\left(x,y\right)$ only represents the baseline terrain of the UAV flight environment, in order to simulate the undulation of the mountainous terrain, we added Equation (28) for representing the detailed data of the mountain slopes in the environment in order to better simulate the UAV flight environment.

(28) $$z_{2}\left(x,y\right)=\sum_{i=1}^{n^{\prime }}\;h_{i}exp\left(-{\left(\frac{x^{2}-x_{i}}{x_{si}}\right)}^{2}-{\left(\frac{y^{2}-y_{i}}{y_{si}}\right)}^{2}\right)$$

where $n^{\prime }$ is the number of hillslopes, $h_{i}$ is the control parameter related to the height of hillslopes, $\left(x_{i},y_{i}\right)$ is the center coordinate of the ith hillslope, and $x_{si}$, $y_{si}$ are the attenuation amount of corresponding hillslopes along the direction of x-axis and y-axis, which are used to represent the specific data of the hills in detail.

#### 5.1.2. Modeling of UAV Flight Cost Function

In this section, to evaluate the paths generated by the algorithm, we assess path quality using four metrics: path length, threat constraints, altitude cost, and flight angle cost. The specific details are as follows:

1. Path Shortest Constraints

The primary goal of UAV path planning is to find a shortest distance between the takeoff point and the target point. In this section, we remember the UAV flight path points as $P_{ij}=(x_{ij},y_{ij},z_{ij})$, i.e., the UAV 3D spatial location of the jth path point in the ith flight path, then the whole flight path $X_{i}$ can be represented as a 3D array containing n path points. Denoting the Euclidean distance between two path points as the path segment $L_{p_{ij}p_{i,j+1}}$, the cost function $F_{1}$ associated with the UAV flight path is represented by Equation (29).

(29) $$F_{1}\left(X_{i}\right)=\sum_{j=1}^{n-1}L_{p_{ij}p_{i,j+1}}$$

2. Threat minimization constraints

In the process of UAV mission, it is inevitable that some obstacles will be encountered in the environment, so we model the obstacles to ensure that the UAV can avoid the obstacles to ensure the safe execution of the mission. In this subsection, we set the obstacle threat area in the form of a cylinder, noting that the cylinder center coordinates are $C_{k}$, the radius is $R_{k}$, and the periphery is the collision threat area D. The obstacle avoidance threat cost of the UAV is inversely proportional to the distance $d_{k}$ between its path segments $p_{ij}p_{i,j+1}$ and the center of the obstacle $C_{k}$. By denoting the set of obstacle threat zones in the flight environment as K and the obstacle threat cost penalty coefficient as Penalty, the cost function $F_{2}$ associated with the UAV obstacle avoidance threat is represented by Equation (30).

(30) $$F_{2}\left(X_{i}\right)=\sum_{j=1}^{n-1}\sum_{k=1}^{K}T_{k}(p_{ij}p_{i,j+1})$$

where $T_{k}\left(p_{ij}p_{i,j+1}\right)$ is given by Equation (31).

(31) $$T_{k}\left(p_{ij}p_{i,j+1}\right)=\left\{\begin{array}{c} 0,\;\;\left(d_{k}\geq D+R_{k}\right)\\ Penalty*\left(D+R_{k}-d_{k}\right),\;\;\left(R_{k}<d_{k}<D+R_{k}\right)\\ \infty ,\;\;(d_{k}\leq R_{k}) \end{array}\right.$$

3. Flight level constraints

The flight altitude of a UAV is usually limited by the constraints of minimum altitude $h_{min}$ and maximum altitude $h_{max}$, where $T_{ij}$ is the terrain altitude and $Z_{ij}$ is the altitude of the UAV. By denoting the height of the UAV from the reference ground at the path point $p_{ij}$ as $h_{ij}$, which is the difference between $Z_{ij}$ and $T_{ij}$, the cost function $H_{ij}$ associated with the current path point $p_{ij}$ of the UAV is represented by Equation (32).

(32) $$H_{ij}=\left\{\begin{array}{c} penalty*(h_{ij}-h_{max}),\;\;\left(h_{ij}\geq h_{max}\right)\\ 0,\;\;\left(h_{min}<h_{ij}<h_{max}\right)\\ penalty*\left(h_{min}-h_{ij}\right),\;\;\left(0<h_{ij}\leq h_{min}\right)\\ \infty ,\;\;(h_{ij}\leq 0) \end{array}\right.$$

meanwhile, by denoting the penalty coefficient for UAV flight altitude exceeding the constraint limitations as penalty, the cost function $F_{3}$ associated with the UAV flight path is represented by Equation (33).

(33) $$F_{3}\left(X_{i}\right)=\sum_{j=1}^{n}H_{ij}$$

4. Flight angle constraints

The UAV flight corner control parameters mainly include horizontal turning angle and vertical pitch angle, which are required in this paper to comply with the actual corner constraint limitations of the UAV, otherwise the trajectory planning model cannot generate feasible flight paths. $L_{p_{ij}p_{i,j+1}}$ and $L_{p_{ij}p_{i,j+2}}$ denote the two consecutive path segments, and $L_{p_{ij}^{\prime }p_{i,j+1}^{\prime }}$ and $L_{p_{ij}^{\prime }p_{i,j+2}^{\prime }}$ are their projections on the xoy plane.

Noting k as the unit vector in the positive direction of the axis, $L_{p_{ij}^{\prime }p_{i,j+1}^{\prime }}$ is calculated by Equation (34). The horizontal turning angle $\alpha_{ij}$ is calculated by Equation (35) and the vertical pitch angle $\beta_{i,j+1}$ is calculated by Equation (36).

(34) $$L_{p_{ij}^{\prime }p_{i,j+1}^{\prime }}=k\times \left(L_{p_{ij}p_{i,j+1}}\times k\right)$$

(35) $$\alpha_{ij}=\arctan\frac{L_{p_{ij}^{\prime }p_{i,j+1}^{\prime }}\times L_{p_{ij}^{\prime }p_{i,j+2}^{\prime }}}{L_{p_{ij}^{\prime }p_{i,j+1}^{\prime }}\cdot L_{p_{ij}^{\prime }p_{i,j+2}^{\prime }}}$$

(36) $$\beta_{ij}=\arctan\frac{z_{i,j+1}-z_{ij}}{∥L_{p_{ij}p_{i,j+1}}∥}$$

Meanwhile, the penalty coefficients for the UAV’s horizontal turning angle and vertical pitch angle exceeding the constraint limitations are denoted as $a_{1}$ and $a_{2}$, respectively, and then the cost function $F_{4}$ associated with the UAV’s flight turning angle is calculated by Equation (37).

(37) $$F_{4}(\begin{array}{c} X_{i}) \end{array}=a_{1}\sum_{j=1}^{n-2}\;\alpha_{ij}+a_{2}\sum_{j=1}^{n-1}\;\left|\beta_{ij}-\beta_{i,j-1}\right|$$

5. Multi-factor flight cost function

Taking into account the shortest path and minimum threat associated with the UAV flight path $X_{i}$, as well as limitations such as flight altitude and flight angle, the flight cost function F based on multifactor constraints is calculated by Equation (38).

(38) $$F(X_{i})=\sum_{k=1}^{4}\;F_{k}(X_{i})/4$$

#### 5.1.3. Analysis of Experimental Results

**Figure 13:**
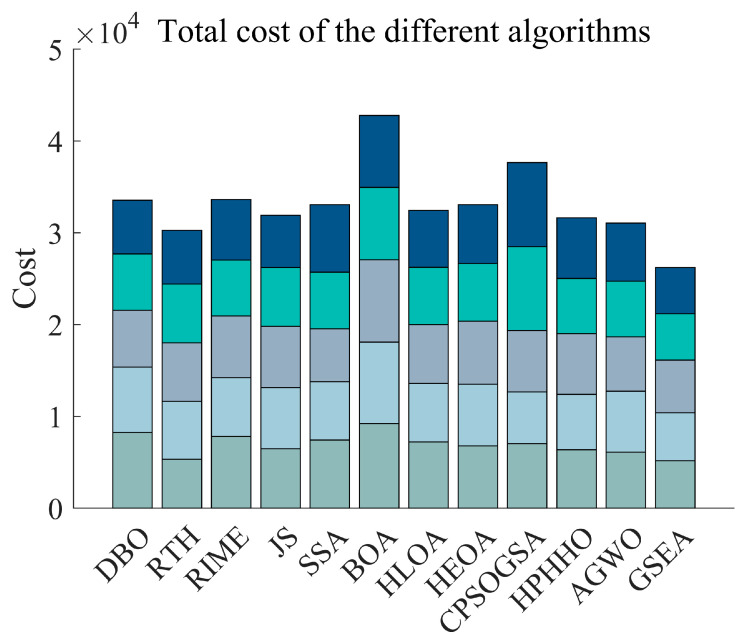
Total cost of planning paths for each algorithm.

**Figure 14:**
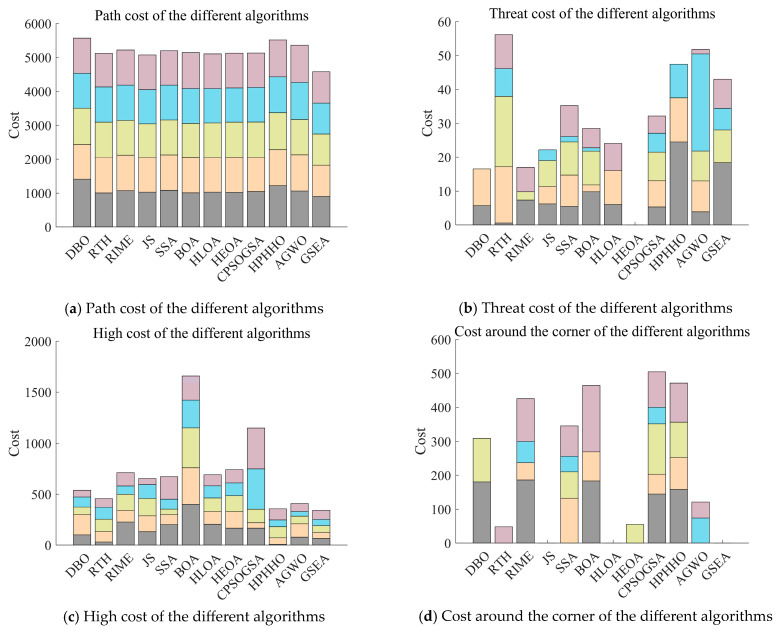
Planning path sub-costs for each algorithm.

**Figure 15:**
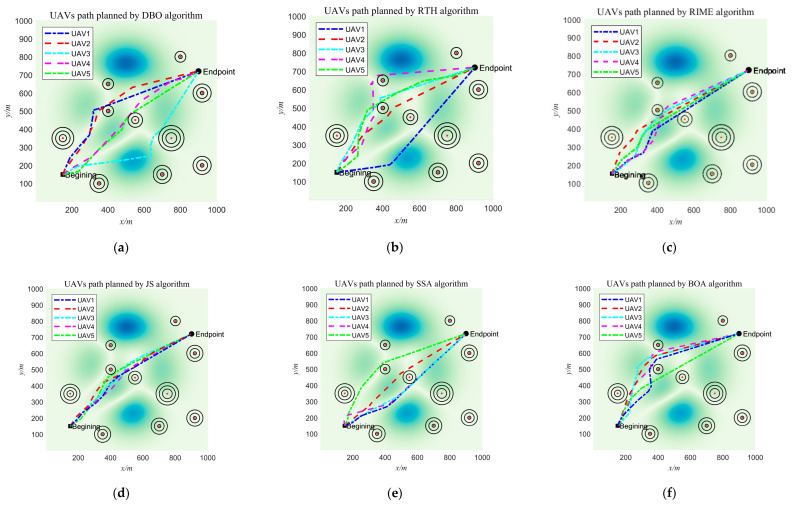

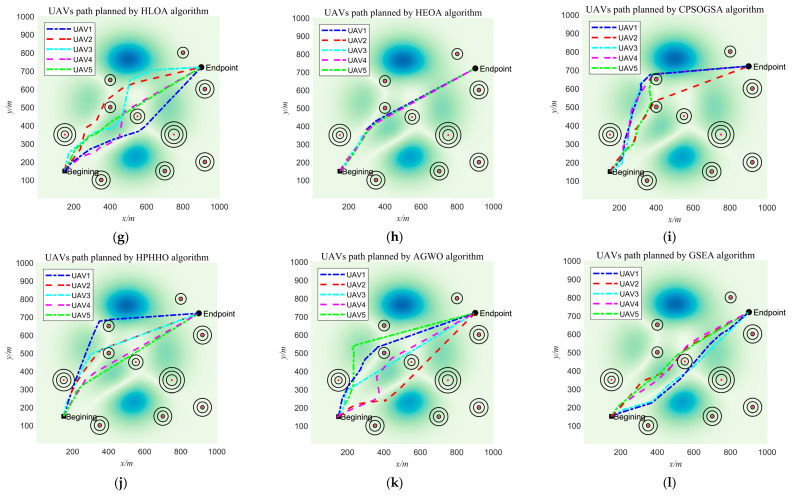
Path planning diagram for each algorithm. (**a**) UAVs path planned by DBO algorithm; (**b**) UAVs path planned by RTH algorithm; (**c**) UAVs path planned by RIME algorithm; (**d**) UAVs path planned by JS algorithm; (**e**) UAVs path planned by SSA algorithm; (**f**) UAVs path planned by BOA algorithm; (**g**) UAVs path planned by HLOA algorithm; (**h**) UAVs path planned by HEOA algorithm; (**i**) UAVs path planned by CPSOGSA algorithm; (**j**) UAVs path planned by HPHHO algorithm; (**k**) UAVs path planned by AGWO algorithm; (**l**) UAVs path planned by GSEA algorithm.

A complete UAV flight environment with threat conditions is obtained through the above modelling process. In this subsection, we perform UAV path planning in the environment obtained from the above modelling. In the experiment, we set the starting point as (150, 150, 50) and the endpoint as (900, 720, 150). In order to have a fair comparison between the competing algorithms, we set the population to 30 and the maximum number of iterations to 1000 for the experiment. Figure 13 shows the total cost of each algorithm for path planning for 5 UAVs in a bar chart. Figure 14 shows the four costs for path, threat, altitude, and corner of the UAVs, respectively. From the figure, GSEA maintains the best total consumed cost results, although in some cases, GSEA does not get the best results. Figure 15 shows the path diagrams planned by each algorithm. It can be seen from the figure that the paths generated by GSEA are smoother than those of the other competing algorithms. This shows the effectiveness and superiority of GSEA for UAV path planning.

From the experimental results, it can be seen that although GSEA did not obtain the best results in terms of some costs, it obtained the best results in terms of the final total cost, ranking first among the 12 algorithms. In future research, we can conduct further studies on these aspects that did not obtain the best results to better improve the performance of the algorithms.

### 5.2. Mobile Robot Path Planning

#### 5.2.1. Environmental Mathematical Model

With the rapid advancements in Internet technology, big data analytics, and artificial intelligence, among other related technologies, mobile robots are poised to revolutionize various industries, including logistics, warehousing, and manufacturing. Path planning, a fundamental technology enabling robots to execute tasks efficiently, has emerged as a prominent research area [74]. To address this challenge, this subsection simulates real-world environments to model robot path planning problems.

In the model, we simulate the robot as a mass for path planning and use the raster method to model the robot’s working map. The raster method consists of binary 0’s and 1’s as the raster values, and the white grid in the map takes the value of 0, indicating that the robot can pass through. The black obstacle grid takes the value of 1, indicating that the robot cannot pass and needs to go around. Cost is a very important factor in judging how well an algorithm plans a path. We design the fitness function based on the robot walking energy consumption to calculate the path cost. One of the most intuitive variables of how much energy a robot consumes to walk is the length of the walking path. We set the robot path consisting of a sequence of points from the start point $\left(x_{0},y_{0}\right)$ to the end point $\left(x_{n},y_{n}\right)$. Therefore, the robot path length is calculated by Equation (39).

(39) $$L=\sum_{i=1}^{n}\sqrt{{\left(x_{i+1}-x_{i}\right)}^{2}-{\left(y_{i+1}-y_{i}\right)}^{2}}$$

From the above, the path cost is calculated by Equation (40).

(40) $$F_{1}=\alpha *L$$

where α is the energy consumption coefficient, and in this paper, we set it to 1 for ease of calculation.

#### 5.2.2. Analysis of the Results of the Dynamic Map Experiment

**Figure 16:**
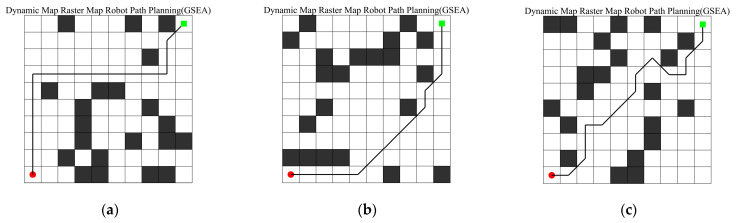

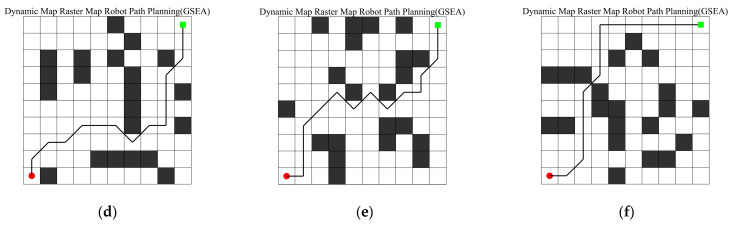
Robot Raster Path Planning Diagram. (**a**) Dynamic Map Raster Map Robot Path Planning (GSEA); (**b**) Dynamic Map Raster Map Robot Path Planning (GSEA); (**c**) Dynamic Map Raster Map Robot Path Planning (GSEA); (**d**) Dynamic Map Raster Map Robot Path Planning (GSEA); (**e**) Dynamic Map Raster Map Robot Path Planning (GSEA); (**f**) Dynamic Map Raster Map Robot Path Planning (GSEA).

This section investigates whether the algorithm can plan paths for the robot under all conditions. We randomize the drone’s grid map by generating black obstacle grids to simulate diverse real-world working environments. Additionally, we set the robot’s starting point to [1, 1] and the endpoint to [10, 10]. During experiments, the robot can move in six directions. GSEA evaluates the quality of each route based on the fitness value for movement in each direction to plan the path. As shown in Figure 16, the GSEA algorithm successfully finds optimal paths for the robot across all six randomized map scenarios, demonstrating its broad applicability.

## 6. Conclusions and Prospects

This paper introduces a novel meta-heuristic algorithm called GSEA inspired by human behaviors, particularly those observed in graduate students. By emulating behaviors such as searching for research directions and focusing on their studies, GSEA demonstrates superior performance across various optimization tasks.

Through extensive experimentation on the CEC2017 and CEC2022 test sets, where GSEA was compared against 11 other popular algorithms, our algorithm consistently achieved the best results. Statistical analyses further confirmed the effectiveness and robustness of GSEA. Moreover, to validate its applicability in real-world scenarios, we applied GSEA to UAV path planning, where it outperformed ten other algorithms. Additionally, when applied to robot path planning across diverse road conditions and randomly generated maps, GSEA consistently produced high-quality paths for the robot. Overall, our findings demonstrate the wide-ranging applicability and effectiveness of the GSEA algorithm across various optimization tasks and real-world problems.

However, in many real-world environments, dynamic threats such as dynamic obstacles often occur, which are not analyzed in this paper and will be a major direction for our future research. In addition, for the establishment of the objective function, we consider it slightly simple; in the future, we will consider multiple factors to be more realistic.

Although GSEA has achieved better results, many areas remain to be explored in future research. In the future, we plan to conduct an in-depth study of GSEA.

1. We plan to combine reinforcement learning with algorithms to enhance the adaptive and self-learning capabilities of the algorithms, so that the algorithms are able to learn while running, and to improve the adaptability of the algorithms in solving problems.
2. We plan to further extend the application area of this algorithm to a wider range of domains, including cloud resource scheduling, fault diagnosis, 3D reconstruction, and so on.

## Figures and Tables

**Table 1:** Descriptions of CEC-2017 benchmark test functions.

No.	Functions	Search Range	Dim	f_min_
Unimodal functions				
F1	Shifted and Rotated Bent Cigar Function	[−100, 100]	30/50/100	100
F3	Shifted and Rotated Zakharov Function	[−100, 100]	30/50/100	300
Simple multimodal functions				
F4	Shifted and Rotated Rosenbrock’s Function	[−100, 100]	30/50/100	400
F5	Shifted and Rotated Rastrigin’s Function	[−100, 100]	30/50/100	500
F6	Shifted and Rotated Expanded Scaffer’s F6 Function	[−100, 100]	30/50/100	600
F7	Shifted and Rotated Lunacek Bi_Rastrigin’s Function	[−100, 100]	30/50/100	700
F8	Shifted and Rotated Non-Continuous Rastrigin’s Function	[−100, 100]	30/50/100	800
F9	Shifted and Rotated Levy Function	[−100, 100]	30/50/100	900
F10	Shifted and Rotated Schwefel’s Function	[−100, 100]	30/50/100	1000
Hybrid functions				
F11	Hybrid Function 1 (N = 3)	[−100, 100]	30/50/100	1100
F12	Hybrid Function 2 (N = 3)	[−100, 100]	30/50/100	1200
F13	Hybrid Function 3 (N = 3)	[−100, 100]	30/50/100	1300
F14	Hybrid Function 4 (N = 4)	[−100, 100]	30/50/100	1400
F15	Hybrid Function 5 (N = 4)	[−100, 100]	30/50/100	1500
F16	Hybrid Function 6 (N = 4)	[−100, 100]	30/50/100	1600
F17	Hybrid Function 6 (N = 5)	[−100, 100]	30/50/100	1700
F18	Hybrid Function 6 (N = 5)	[−100, 100]	30/50/100	1800
F19	Hybrid Function 6 (N = 5)	[−100, 100]	30/50/100	1900
F20	Hybrid Function 6 (N = 6)	[−100, 100]	30/50/100	2000
Composition functions				
F21	Composition Function 1 (N = 3)	[−100, 100]	30/50/100	2100
F22	Composition Function 2 (N = 3)	[−100, 100]	30/50/100	2200
F23	Composition Function 3 (N = 4)	[−100, 100]	30/50/100	2300
F24	Composition Function 4 (N = 4)	[−100, 100]	30/50/100	2400
F25	Composition Function 5 (N = 5)	[−100, 100]	30/50/100	2500
F26	Composition Function 6 (N = 5)	[−100, 100]	30/50/100	2600
F27	Composition Function 7 (N = 6)	[−100, 100]	30/50/100	2700
F28	Composition Function 8 (N = 6)	[−100, 100]	30/50/100	2800
F29	Composition Function 9 (N = 3)	[−100, 100]	30/50/100	2900
F30	Composition Function 10 (N = 3)	[−100, 100]	30/50/100	3000

**Table 2:** Descriptions of CEC-2022 benchmark test functions.

No.	Functions	Search Range	Dim	f_min_
Unimodal function				
F1	Shifted and full Rotated Zakharov Function	[−100, 100]	20	300
Basic Functions				
F2	Shifted and full Rotated Rosenbrock’s Function	[−100, 100]	20	400
F3	Shifted and full Expanded Schaffer’s f6 Function	[−100, 100]	20	600
F4	Shifted and full Rotated Non-Continuous Rastrigin’s Function	[−100, 100]	20	800
F5	Shifted and full Rotated Levy Function	[−100, 100]	20	900
Hybrid functions				
F6	Hybrid Function 1 (N = 3)	[−100, 100]	20	1800
F7	Hybrid Function 2 (N = 6)	[−100, 100]	20	2000
F8	Hybrid Function 3 (N = 5)	[−100, 100]	20	2200
Composition functions				
F9	Composition Function 1 (N = 5)	[−100, 100]	20	2300
F10	Composition Function 2 (N = 4)	[−100, 100]	20	2400
F11	Composition Function 3 (N = 5)	[−100, 100]	20	2600
F12	Composition Function 5 (N = 6)	[−100, 100]	20	2700

**Table 3:** Parameter settings of the comparison algorithms.

Algorithm	Name of the Parameter	Value of the Parameter	n	T	References
DBO	P_percent	0.2	30	1000	[59]
RTH	A , r $,\;R_{0}$	15, 1.5, 0.5	30	1000	[60]
RIME	W	5	30	1000	[61]
JS	tt	1	30	1000	[62]
SSA	P	0.2	30	1000	[63]
BOA	p	0.8	30	1000	[64]
HLOA	w	0.5	30	1000	[65]
HEOA	A , LN , EN , FN	0.6, 0.4, 0.4, 0.1	30	1000	[66]
CPSOGSA	phi1 , phi2	2.05, 2.05	30	1000	[67]
HPHHO	F , CR , λ , β , J	0.5, 0.9, 0.5, 1.5, [0, 2]	30	1000	[68]
AGWO	V , l	0.8, 0.5	30	1000	[69]

**Table 4:** Experimental results of the state-of-the-art algorithms on the CEC 2017 (dim = 30).

ID	Items	DBO	RTH	RIME	JS	SSA	BOA	HLOA	HEOA	CPSOGSA	HPHHO	AGWO	GSEA
CEC2017-F1	Ave	3.5917E+07	6.1849E+03	6.0357E+05	6.9582E+05	6.5229E+03	4.9600E+10	4.6001E+07	1.2816E+10	3.4255E+05	1.2277E+09	1.2039E+10	4.0225E+02
	Std	3.2062E+07	6.6127E+03	2.2575E+05	1.6078E+06	7.2584E+03	6.9918E+09	2.6820E+07	4.6810E+09	1.7892E+06	5.5768E+08	2.9583E+09	2.8337E+02
CEC2017-F2	Ave	6.6797E+31	2.7192E+18	3.7714E+13	6.5759E+17	1.0930E+13	6.1868E+53	1.6693E+37	1.1474E+41	1.1034E+30	1.0000E+20	2.3096E+35	9.7396E+08
	Std	3.6241E+32	1.4771E+19	6.5857E+13	2.1034E+18	2.3375E+13	3.2655E+54	7.2150E+37	6.2847E+41	2.8391E+30	0.0000E+00	1.0855E+36	1.7532E+09
CEC2017-F3	Ave	7.4586E+04	3.0000E+02	1.2443E+04	4.1394E+04	2.9344E+04	7.8200E+04	2.0451E+04	6.5972E+04	1.1454E+05	3.6085E+04	6.5946E+04	8.5211E+03
	Std	1.2693E+04	1.3717E-03	3.8797E+03	7.8544E+03	5.5459E+03	6.6435E+03	7.7203E+03	7.3365E+03	3.8327E+04	9.2223E+03	1.0467E+04	2.3263E+03
CEC2017-F4	Ave	5.9364E+02	4.4586E+02	5.0961E+02	5.2508E+02	4.9426E+02	2.1605E+04	5.5261E+02	1.6348E+03	5.1909E+02	6.6115E+02	1.5416E+03	4.6809E+02
	Std	8.2621E+01	3.5587E+01	2.5032E+01	3.0601E+01	2.6146E+01	3.2577E+03	4.4405E+01	6.1414E+02	3.9820E+01	1.0991E+02	7.0115E+02	2.7255E+01
CEC2017-F5	Ave	7.5526E+02	6.8351E+02	5.9539E+02	5.8809E+02	7.5090E+02	9.1177E+02	8.0822E+02	8.4022E+02	8.0885E+02	7.5711E+02	7.7489E+02	5.6961E+02
	Std	6.1758E+01	3.4393E+01	2.3437E+01	2.3026E+01	5.6826E+01	2.3200E+01	4.4307E+01	3.8428E+01	5.0587E+01	3.7713E+01	3.1465E+01	8.3017E+00
CEC2017-F6	Ave	6.5089E+02	6.4137E+02	6.0908E+02	6.1478E+02	6.4483E+02	6.8593E+02	6.7477E+02	6.7251E+02	6.6710E+02	6.5787E+02	6.5869E+02	6.0056E+02
	Std	9.0743E+00	8.1505E+00	5.5324E+00	4.4188E+00	1.2300E+01	7.2815E+00	1.0748E+01	7.1028E+00	7.9285E+00	6.7616E+00	7.5579E+00	3.3184E-01
CEC2017-F7	Ave	1.0015E+03	1.0978E+03	8.3888E+02	8.7526E+02	1.2550E+03	1.3826E+03	1.3348E+03	1.3572E+03	1.5970E+03	1.2013E+03	1.1168E+03	8.3243E+02
	Std	9.0008E+01	8.7582E+01	2.8198E+01	5.0293E+01	8.4973E+01	3.7165E+01	4.9569E+01	8.0332E+01	2.2772E+02	9.8517E+01	4.5043E+01	1.2611E+01
CEC2017-F8	Ave	1.0332E+03	9.4357E+02	9.0109E+02	8.7773E+02	9.7130E+02	1.1397E+03	1.0047E+03	1.0820E+03	1.0210E+03	9.9495E+02	1.0150E+03	8.6552E+02
	Std	5.3794E+01	3.1697E+01	3.1601E+01	2.2869E+01	2.9512E+01	2.1955E+01	3.6856E+01	2.9766E+01	4.2480E+01	2.2913E+01	2.5096E+01	8.3413E+00
CEC2017-F9	Ave	5.6889E+03	4.1045E+03	2.7125E+03	1.8943E+03	5.1297E+03	1.1171E+04	6.7049E+03	8.1965E+03	7.4810E+03	6.1328E+03	6.5537E+03	1.1884E+03
	Std	1.7680E+03	6.5502E+02	1.5904E+03	6.9700E+02	5.7480E+02	1.0251E+03	1.0567E+03	1.3461E+03	1.8736E+03	5.6936E+02	1.0645E+03	1.2470E+02
CEC2017-F10	Ave	6.4907E+03	5.2775E+03	4.2618E+03	8.1459E+03	5.2330E+03	9.0118E+03	6.6383E+03	7.4159E+03	5.1057E+03	5.6050E+03	7.1174E+03	3.8609E+03
	Std	9.8029E+02	6.4865E+02	6.1824E+02	6.1961E+02	6.3391E+02	3.2088E+02	9.2365E+02	6.3230E+02	6.6812E+02	6.1456E+02	8.7168E+02	4.1218E+02
CEC2017-F11	Ave	1.5945E+03	1.2438E+03	1.2762E+03	1.2294E+03	1.3100E+03	7.6172E+03	1.3846E+03	3.8948E+03	1.2639E+03	1.4813E+03	4.1183E+03	1.1383E+03
	Std	2.6798E+02	4.7806E+01	4.7345E+01	3.7960E+01	6.8238E+01	2.1249E+03	7.1983E+01	1.2975E+03	5.6351E+01	1.0429E+02	1.7348E+03	1.4089E+01
CEC2017-F12	Ave	4.7778E+07	3.2377E+04	1.1018E+07	1.1476E+06	6.6517E+05	1.3761E+10	1.8383E+07	3.2692E+08	3.1597E+06	8.5914E+07	5.7649E+08	1.3743E+05
	Std	7.4876E+07	1.5838E+04	1.0673E+07	9.3309E+05	5.4980E+05	4.1771E+09	1.6878E+07	2.0065E+08	4.1918E+06	5.7737E+07	4.4606E+08	6.8600E+04
CEC2017-F13	Ave	4.5835E+06	2.0645E+04	6.2328E+04	1.0369E+04	1.6544E+05	1.1639E+10	7.7669E+05	5.5986E+06	5.4152E+04	1.1039E+06	1.1234E+08	3.1487E+03
	Std	7.9353E+06	2.2344E+04	8.5956E+04	9.6460E+03	8.1151E+05	4.7243E+09	1.7000E+06	1.1422E+07	2.9687E+04	1.3036E+06	1.9572E+08	1.3943E+03
CEC2017-F14	Ave	1.8232E+05	2.1765E+03	6.5564E+04	1.6559E+04	4.3685E+04	4.0150E+06	1.0391E+05	1.1401E+06	8.9122E+04	3.4026E+05	7.9721E+05	3.8242E+03
	Std	2.2334E+05	6.6556E+02	6.0435E+04	1.4086E+04	3.1281E+04	3.1546E+06	1.5342E+05	8.6343E+05	1.1784E+05	3.3438E+05	7.9360E+05	1.0997E+03
CEC2017-F15	Ave	9.3324E+04	9.6126E+03	1.2122E+04	5.7000E+03	1.2874E+04	5.4226E+08	1.2495E+05	6.7504E+05	1.9950E+04	4.1997E+04	2.2551E+06	2.8888E+03
	Std	8.5410E+04	1.1606E+04	1.1084E+04	4.0784E+03	1.6466E+04	4.2597E+08	5.2038E+05	7.1902E+05	1.2791E+04	3.4167E+04	3.7172E+06	9.6464E+02
CEC2017-F16	Ave	3.4018E+03	2.8265E+03	2.6325E+03	2.4724E+03	2.9970E+03	7.6177E+03	3.8453E+03	3.7639E+03	3.2760E+03	3.0066E+03	3.3388E+03	2.1513E+03
	Std	4.0297E+02	2.9317E+02	2.7966E+02	2.7851E+02	3.4795E+02	2.4171E+03	6.6316E+02	8.3133E+02	3.4030E+02	4.1709E+02	3.0521E+02	1.6421E+02
CEC2017-F17	Ave	2.5946E+03	2.4975E+03	2.1263E+03	1.9309E+03	2.5055E+03	1.3328E+04	2.9229E+03	2.6109E+03	2.6344E+03	2.3552E+03	2.3982E+03	1.8188E+03
	Std	3.1361E+02	2.8548E+02	2.2926E+02	1.2367E+02	2.9816E+02	1.8505E+04	3.4021E+02	2.8533E+02	2.6746E+02	2.6904E+02	2.5625E+02	6.3493E+01
CEC2017-F18	Ave	3.3070E+06	2.5114E+04	8.0717E+05	6.3591E+05	3.3263E+05	6.9863E+07	5.5877E+05	6.0215E+06	9.0152E+05	1.4525E+06	5.1933E+06	5.3281E+04
	Std	4.4027E+06	3.3420E+04	5.9211E+05	6.8027E+05	3.0403E+05	9.1958E+07	5.9755E+05	5.1486E+06	1.0288E+06	1.1923E+06	7.7429E+06	2.2037E+04
CEC2017-F19	Ave	5.7797E+06	7.0364E+03	2.0534E+04	7.8626E+03	9.9326E+03	5.1978E+08	6.4581E+04	5.3768E+06	1.2615E+04	1.1514E+06	1.0284E+07	2.3419E+03
	Std	1.8557E+07	6.4845E+03	1.9772E+04	6.4607E+03	1.3840E+04	3.5941E+08	8.9073E+04	2.8668E+06	1.4688E+04	1.8548E+06	2.6679E+07	3.6972E+02
CEC2017-F20	Ave	2.6207E+03	2.6932E+03	2.4926E+03	2.3648E+03	2.7817E+03	3.0188E+03	3.0744E+03	2.8113E+03	2.9834E+03	2.5838E+03	2.7129E+03	2.1342E+03
	Std	1.9840E+02	2.1949E+02	2.0727E+02	1.3261E+02	1.8385E+02	1.2865E+02	2.7287E+02	2.1902E+02	2.5557E+02	1.8983E+02	1.9281E+02	4.9219E+01
CEC2017-F21	Ave	2.5673E+03	2.4647E+03	2.4019E+03	2.3754E+03	2.5150E+03	2.7161E+03	2.6546E+03	2.5906E+03	2.5663E+03	2.5318E+03	2.5541E+03	2.3598E+03
	Std	4.5157E+01	3.9287E+01	3.2599E+01	1.7421E+01	3.9573E+01	6.6462E+01	5.6610E+01	3.4566E+01	4.1545E+01	4.0631E+01	2.9194E+01	8.3140E+00
CEC2017-F22	Ave	5.0790E+03	4.5211E+03	4.8287E+03	2.3116E+03	6.0563E+03	6.9834E+03	7.8902E+03	7.3159E+03	6.5842E+03	4.8203E+03	6.1318E+03	2.3000E+03
	Std	2.2161E+03	2.3253E+03	1.9119E+03	8.5503E+00	2.0360E+03	1.0948E+03	1.5992E+03	1.8105E+03	1.3761E+03	2.5442E+03	2.4781E+03	1.6707E-07
CEC2017-F23	Ave	3.0296E+03	2.8944E+03	2.7731E+03	2.7889E+03	2.9384E+03	3.4644E+03	3.3652E+03	3.1291E+03	3.1166E+03	2.9709E+03	3.0236E+03	2.7012E+03
	Std	6.1993E+01	6.3111E+01	3.1507E+01	4.0008E+01	7.4832E+01	1.5564E+02	1.6653E+02	1.3445E+02	9.5442E+01	8.6136E+01	6.2097E+01	1.1623E+01
CEC2017-F24	Ave	3.1734E+03	3.0416E+03	2.9306E+03	2.9594E+03	3.0736E+03	4.0549E+03	3.5411E+03	3.1954E+03	3.3024E+03	3.1792E+03	3.2496E+03	2.8724E+03
	Std	6.7364E+01	8.1154E+01	2.7556E+01	3.8451E+01	8.2529E+01	2.5318E+02	1.9413E+02	8.5994E+01	8.9352E+01	9.1974E+01	3.8932E+01	8.1961E+00
CEC2017-F25	Ave	2.9523E+03	2.8974E+03	2.9048E+03	2.9311E+03	2.9024E+03	5.7927E+03	2.9785E+03	3.2093E+03	2.9302E+03	3.0373E+03	3.1781E+03	2.8855E+03
	Std	4.7761E+01	1.5562E+01	2.4442E+01	2.7927E+01	2.0983E+01	5.1615E+02	3.6628E+01	7.7617E+01	2.8245E+01	3.4916E+01	6.9337E+01	1.7607E+00
CEC2017-F26	Ave	6.7026E+03	5.4904E+03	4.7558E+03	4.7805E+03	6.2757E+03	1.1801E+04	9.0860E+03	8.3385E+03	7.6901E+03	6.7037E+03	7.3690E+03	4.2848E+03
	Std	1.1183E+03	1.6497E+03	6.1238E+02	1.3148E+03	1.1782E+03	7.7475E+02	1.3958E+03	1.0271E+03	9.1567E+02	1.4655E+03	8.7087E+02	6.0534E+02
CEC2017-F27	Ave	3.3558E+03	3.2661E+03	3.2366E+03	3.2944E+03	3.2864E+03	4.2856E+03	3.7557E+03	3.4462E+03	3.5855E+03	3.3434E+03	3.4654E+03	3.2191E+03
	Std	8.5092E+01	4.5819E+01	1.3575E+01	2.9849E+01	6.1784E+01	3.2111E+02	3.4559E+02	1.0326E+02	3.0558E+02	7.7061E+01	8.7042E+01	5.0881E+00
CEC2017-F28	Ave	3.4628E+03	3.1452E+03	3.2732E+03	3.3129E+03	3.2078E+03	8.2799E+03	3.3093E+03	4.1391E+03	3.2688E+03	3.4136E+03	3.8814E+03	3.2038E+03
	Std	2.9725E+02	5.9412E+01	5.3291E+01	3.6568E+01	2.8021E+01	4.8780E+02	3.1389E+01	3.2287E+02	2.7164E+01	6.8985E+01	2.1212E+02	4.6917E+00
CEC2017-F29	Ave	4.5364E+03	4.2239E+03	3.9469E+03	3.8814E+03	4.1996E+03	1.5177E+04	5.8414E+03	5.3726E+03	4.5657E+03	4.3456E+03	4.6519E+03	3.4934E+03
	Std	3.4669E+02	4.1072E+02	2.5985E+02	2.0379E+02	3.1400E+02	1.1299E+04	8.0940E+02	4.7225E+02	3.2454E+02	3.1564E+02	2.7251E+02	8.9152E+01
CEC2017-F30	Ave	4.6413E+06	1.0560E+04	2.5265E+05	1.1828E+04	5.2803E+04	1.7058E+09	1.2929E+06	5.4702E+07	3.0454E+05	9.5511E+06	3.6086E+07	6.4192E+03
	Std	6.1151E+06	3.4820E+03	2.4365E+05	6.0650E+03	2.1211E+05	9.7107E+08	1.5223E+06	3.2007E+07	2.8669E+05	8.6288E+06	2.5334E+07	4.2602E+02

**Table 5:** Experimental results of the state-of-the-art algorithms on the CEC 2017 (dim = 50).

ID	Items	DBO	RTH	RIME	JS	SSA	BOA	HLOA	HEOA	CPSOGSA	HPHHO	AGWO	GSEA
CEC2017-F1	Ave	1.0352E+09	3.2161E+03	7.1385E+06	3.9286E+08	3.1403E+03	1.0622E+11	4.2916E+08	4.0038E+10	1.4358E+09	1.5493E+10	3.6850E+10	4.4609E+02
	Std	9.3957E+08	3.9047E+03	2.1754E+06	4.1611E+08	2.8676E+03	8.3616E+09	2.8012E+08	1.1552E+10	5.7344E+09	3.5209E+09	6.7610E+09	1.9159E+02
CEC2017-F2	Ave	3.2029E+63	2.9854E+39	2.0236E+34	1.6378E+48	1.9626E+40	3.4676E+95	7.4192E+70	1.7139E+66	7.1803E+66	1.0000E+20	3.1678E+62	4.3605E+26
	Std	9.6579E+63	1.6219E+40	1.0203E+35	8.9589E+48	1.0749E+41	1.8990E+96	3.1499E+71	7.8659E+66	2.9251E+67	0.0000E+00	1.5725E+63	8.0303E+26
CEC2017-F3	Ave	2.1568E+05	5.5802E+03	1.2470E+05	1.4889E+05	1.7460E+05	2.8095E+05	1.3525E+05	1.7482E+05	3.0591E+05	1.0549E+05	1.4787E+05	6.1603E+04
	Std	4.2777E+04	3.5797E+03	2.9843E+04	1.9991E+04	3.4714E+04	8.8415E+04	3.8760E+04	1.3825E+04	7.4187E+04	1.2338E+04	1.6639E+04	7.7734E+03
CEC2017-F4	Ave	9.1675E+02	5.0510E+02	6.5293E+02	7.7879E+02	5.5288E+02	4.0188E+04	8.1116E+02	6.9184E+03	8.1777E+02	2.0776E+03	6.8747E+03	4.8824E+02
	Std	2.3022E+02	5.4255E+01	4.8902E+01	1.2080E+02	5.8859E+01	4.3740E+03	8.0391E+01	2.8567E+03	3.6449E+02	6.6221E+02	1.7430E+03	2.2595E+01
CEC2017-F5	Ave	9.5303E+02	8.3198E+02	7.2401E+02	7.0518E+02	8.7615E+02	1.1765E+03	9.6972E+02	1.1150E+03	1.0397E+03	9.5410E+02	1.0218E+03	6.7779E+02
	Std	1.0843E+02	4.0545E+01	3.9602E+01	4.0864E+01	2.1345E+01	2.5705E+01	5.4718E+01	3.4069E+01	7.0686E+01	3.0949E+01	3.6743E+01	2.3191E+01
CEC2017-F6	Ave	6.6504E+02	6.5160E+02	6.2203E+02	6.2785E+02	6.6165E+02	7.0143E+02	6.8070E+02	6.9013E+02	6.7580E+02	6.7246E+02	6.7840E+02	6.0643E+02
	Std	9.7807E+00	6.0098E+00	6.2915E+00	6.2143E+00	6.1545E+00	5.9034E+00	5.2271E+00	5.5256E+00	7.7145E+00	6.2558E+00	6.8335E+00	1.4370E+00
CEC2017-F7	Ave	1.3740E+03	1.5154E+03	1.0423E+03	1.1650E+03	1.7255E+03	2.0016E+03	1.8648E+03	1.9847E+03	2.6491E+03	1.7626E+03	1.5560E+03	1.0410E+03
	Std	1.2956E+02	1.1950E+02	5.3130E+01	9.6066E+01	6.7790E+01	4.4162E+01	1.0374E+02	5.9994E+01	3.7037E+02	8.5481E+01	9.1653E+01	3.7898E+01
CEC2017-F8	Ave	1.2973E+03	1.1368E+03	1.0153E+03	1.0218E+03	1.2008E+03	1.4982E+03	1.3115E+03	1.4356E+03	1.2469E+03	1.2398E+03	1.3435E+03	9.8620E+02
	Std	1.0933E+02	5.6533E+01	4.1938E+01	5.0005E+01	2.7193E+01	2.3887E+01	5.6435E+01	5.2063E+01	6.0933E+01	3.3870E+01	3.1327E+01	2.5278E+01
CEC2017-F9	Ave	2.4320E+04	1.1296E+04	8.3007E+03	1.1234E+04	1.3236E+04	3.7858E+04	2.0464E+04	2.7827E+04	2.0335E+04	2.0881E+04	2.8482E+04	3.4789E+03
	Std	7.5348E+03	1.4133E+03	2.5273E+03	3.4037E+03	9.5044E+02	3.1599E+03	2.5714E+03	2.5525E+03	2.8774E+03	2.8641E+03	4.3008E+03	5.1594E+02
CEC2017-F10	Ave	1.0504E+04	7.8925E+03	7.7969E+03	1.4483E+04	8.5374E+03	1.5417E+04	1.2199E+04	1.2730E+04	8.5482E+03	1.0091E+04	1.2737E+04	6.2196E+03
	Std	2.3513E+03	8.6017E+02	9.0606E+02	1.1231E+03	9.7740E+02	5.8959E+02	1.7146E+03	1.0735E+03	9.9953E+02	9.9906E+02	1.0379E+03	4.5311E+02
CEC2017-F11	Ave	2.7119E+03	1.3451E+03	1.5745E+03	1.4777E+03	1.3396E+03	2.4730E+04	1.8778E+03	7.5888E+03	2.2804E+03	2.6504E+03	1.1051E+04	1.2171E+03
	Std	1.8394E+03	5.8714E+01	6.9800E+01	1.2607E+02	6.7504E+01	2.2268E+03	2.3543E+02	2.4675E+03	8.9118E+02	7.3693E+02	2.9820E+03	1.7055E+01
CEC2017-F12	Ave	4.8326E+08	5.8183E+05	8.9582E+07	1.2793E+07	5.4751E+06	7.5060E+10	2.3138E+08	7.1195E+09	7.9147E+07	1.0887E+09	1.0649E+10	1.2731E+06
	Std	3.9342E+08	3.1466E+05	5.5210E+07	7.4613E+06	3.2971E+06	1.7716E+10	1.2616E+08	4.3108E+09	7.1754E+07	6.6137E+08	4.1960E+09	3.6383E+05
CEC2017-F13	Ave	3.1255E+07	1.2317E+04	2.2464E+05	1.1294E+04	3.1074E+04	4.6553E+10	5.2266E+06	2.8933E+08	3.4393E+07	6.8029E+07	1.2549E+09	1.7241E+03
	Std	4.0098E+07	1.1358E+04	1.1961E+05	4.0974E+03	2.2281E+04	1.5421E+10	1.1219E+07	2.6016E+08	1.4323E+08	5.5911E+07	1.6585E+09	1.5837E+02
CEC2017-F14	Ave	3.3000E+06	1.2958E+04	3.2793E+05	1.0941E+05	1.8649E+05	1.1641E+08	8.3068E+05	4.4256E+06	4.3093E+05	1.5483E+06	6.4363E+06	3.3864E+04
	Std	3.3751E+06	1.1567E+04	1.7036E+05	7.3093E+04	9.3692E+04	9.7642E+07	6.3074E+05	4.0197E+06	4.6680E+05	1.2849E+06	6.7554E+06	1.5060E+04
CEC2017-F15	Ave	1.0960E+07	1.3334E+04	6.9838E+04	8.1999E+03	1.8482E+04	8.0067E+09	6.3597E+04	1.3699E+08	4.9566E+04	2.7621E+06	3.3705E+08	2.7232E+03
	Std	2.4443E+07	8.0469E+03	6.7527E+04	5.8804E+03	9.3018E+03	3.0972E+09	5.2037E+04	2.9544E+08	2.9908E+04	2.5022E+06	3.7444E+08	1.0236E+03
CEC2017-F16	Ave	4.6348E+03	3.6274E+03	3.6090E+03	3.1898E+03	3.9060E+03	1.0713E+04	5.1683E+03	5.7867E+03	4.1334E+03	4.4630E+03	4.5154E+03	2.7306E+03
	Std	6.0851E+02	5.3328E+02	4.1176E+02	3.8306E+02	5.8464E+02	1.6169E+03	9.3909E+02	1.0480E+03	5.1136E+02	6.0936E+02	3.6187E+02	1.7466E+02
CEC2017-F17	Ave	4.1503E+03	3.5704E+03	3.2972E+03	3.0422E+03	3.5216E+03	1.5537E+04	4.2942E+03	4.1595E+03	3.6957E+03	3.6251E+03	3.8077E+03	2.5426E+03
	Std	5.0739E+02	3.5587E+02	3.0186E+02	3.1803E+02	4.1516E+02	6.3220E+03	4.7755E+02	8.0323E+02	4.2740E+02	3.2539E+02	3.4347E+02	1.3471E+02
CEC2017-F18	Ave	5.8481E+06	8.2453E+04	4.1977E+06	1.2855E+06	1.6793E+06	1.9793E+08	3.7043E+06	4.0702E+07	1.9157E+06	5.6233E+06	2.6131E+07	3.4990E+05
	Std	5.7943E+06	6.3640E+04	3.2709E+06	8.0108E+05	1.1048E+06	1.2105E+08	3.0243E+06	2.6595E+07	1.6006E+06	5.3235E+06	2.2625E+07	1.2933E+05
CEC2017-F19	Ave	6.7889E+06	1.6356E+04	5.7468E+04	2.0330E+04	1.8006E+04	3.5071E+09	6.1812E+05	4.6626E+07	6.2080E+04	1.9101E+06	9.8785E+07	1.5215E+04
	Std	1.2886E+07	9.5971E+03	6.8413E+04	9.0598E+03	1.4015E+04	2.0846E+09	6.5308E+05	6.4513E+07	7.2921E+04	1.7683E+06	1.3836E+08	3.9835E+03
CEC2017-F20	Ave	3.6740E+03	3.3841E+03	3.1957E+03	3.5108E+03	3.5472E+03	4.2816E+03	4.0122E+03	3.7994E+03	3.4803E+03	3.3138E+03	3.5047E+03	2.6304E+03
	Std	3.3425E+02	3.0516E+02	3.1522E+02	4.0165E+02	3.7616E+02	1.8820E+02	4.1729E+02	2.5299E+02	2.7657E+02	2.3818E+02	2.4491E+02	8.8962E+01
CEC2017-F21	Ave	2.8693E+03	2.6489E+03	2.5292E+03	2.4797E+03	2.7645E+03	3.2051E+03	3.0519E+03	2.9390E+03	2.9432E+03	2.8309E+03	2.8580E+03	2.4428E+03
	Std	9.0938E+01	8.3208E+01	3.8230E+01	4.6725E+01	8.9440E+01	6.3510E+01	1.4251E+02	7.6021E+01	1.0086E+02	5.1720E+01	4.2587E+01	1.2042E+01
CEC2017-F22	Ave	1.2699E+04	1.0026E+04	9.3826E+03	1.5073E+04	1.0328E+04	1.7066E+04	1.3782E+04	1.3569E+04	1.0347E+04	1.2190E+04	1.5565E+04	7.2353E+03
	Std	2.1914E+03	1.0156E+03	9.5927E+02	2.8653E+03	9.9450E+02	5.9999E+02	1.2246E+03	1.1354E+03	9.7496E+02	1.1287E+03	9.2705E+02	2.0428E+03
CEC2017-F23	Ave	3.4903E+03	3.2556E+03	3.0043E+03	3.1005E+03	3.3268E+03	4.5273E+03	4.2223E+03	3.6917E+03	3.6133E+03	3.4506E+03	3.6239E+03	2.8783E+03
	Std	1.9103E+02	1.2819E+02	5.5002E+01	6.7197E+01	1.2897E+02	2.2521E+02	3.3075E+02	1.5214E+02	1.5027E+02	1.1162E+02	9.1596E+01	2.0576E+01
CEC2017-F24	Ave	3.6902E+03	3.4589E+03	3.1400E+03	3.2842E+03	3.4945E+03	5.3011E+03	4.2858E+03	3.7587E+03	3.9791E+03	3.6822E+03	3.8826E+03	3.0336E+03
	Std	1.5132E+02	1.3762E+02	5.5073E+01	6.1681E+01	1.3687E+02	2.1638E+02	3.2067E+02	1.5259E+02	1.3753E+02	1.0555E+02	1.0000E+02	1.3021E+01
CEC2017-F25	Ave	3.4477E+03	3.0490E+03	3.1224E+03	3.2814E+03	3.0708E+03	1.6107E+04	3.2646E+03	6.6959E+03	3.1638E+03	4.1441E+03	5.6631E+03	3.0842E+03
	Std	8.2391E+02	3.5363E+01	4.6769E+01	6.8841E+01	3.4888E+01	1.1315E+03	8.0439E+01	1.0450E+03	5.1007E+01	3.9994E+02	6.7586E+02	1.5704E+01
CEC2017-F26	Ave	9.5904E+03	9.2908E+03	6.5900E+03	8.9797E+03	6.5277E+03	1.7662E+04	1.3578E+04	1.3923E+04	1.2779E+04	1.1563E+04	1.2352E+04	6.3971E+03
	Std	2.0118E+03	2.8295E+03	4.0099E+02	1.1139E+03	3.7663E+03	6.4046E+02	1.3468E+03	8.8759E+02	1.3113E+03	1.8854E+03	9.9242E+02	6.2839E+02
CEC2017-F27	Ave	4.0151E+03	3.6802E+03	3.5969E+03	3.8349E+03	3.6655E+03	6.6795E+03	4.9700E+03	4.3278E+03	4.8187E+03	3.9428E+03	4.9153E+03	3.4169E+03
	Std	2.0410E+02	2.1842E+02	1.0596E+02	1.4625E+02	1.8132E+02	8.1138E+02	6.4202E+02	4.5814E+02	4.5829E+02	2.2137E+02	2.6956E+02	3.6372E+01
CEC2017-F28	Ave	5.7445E+03	3.2917E+03	3.3748E+03	3.8343E+03	3.3245E+03	1.4639E+04	3.5839E+03	6.6665E+03	3.6776E+03	4.7988E+03	6.6848E+03	3.3345E+03
	Std	2.2784E+03	2.2899E+01	3.6776E+01	1.8664E+02	3.3998E+01	1.2293E+03	1.1752E+02	6.7297E+02	4.4803E+02	3.5689E+02	6.9041E+02	1.7773E+01
CEC2017-F29	Ave	6.3772E+03	4.9379E+03	4.8263E+03	4.6316E+03	5.0490E+03	4.0237E+05	8.4154E+03	8.4488E+03	6.4267E+03	6.4179E+03	7.4581E+03	3.8256E+03
	Std	1.1055E+03	5.2502E+02	3.5890E+02	3.3468E+02	5.0886E+02	3.0695E+05	1.0611E+03	1.3692E+03	7.4359E+02	8.6325E+02	5.9816E+02	1.5359E+02
CEC2017-F30	Ave	3.8667E+07	9.5036E+05	3.1747E+07	4.1280E+06	1.6750E+06	6.8739E+09	8.7219E+07	4.0034E+08	5.9862E+07	1.7739E+08	3.7760E+08	8.1160E+05
	Std	4.7184E+07	2.2367E+05	1.4933E+07	1.7022E+06	8.6338E+05	2.8884E+09	5.3803E+07	1.9373E+08	1.6879E+07	7.7781E+07	2.0162E+08	4.4879E+04

**Table 6:** Experimental results of the state-of-the-art algorithms on the CEC 2017 (dim = 100).

ID	Items	DBO	RTH	RIME	JS	SSA	BOA	HLOA	HEOA	CPSOGSA	HPHHO	AGWO	GSEA
CEC2017-F1	Ave	3.7741E+10	9.3622E+03	1.6095E+08	2.2829E+10	4.3604E+06	2.5971E+11	4.6850E+09	1.5313E+11	8.9245E+09	8.1796E+10	1.5065E+11	5.1680E+07
	Std	4.7516E+10	9.2640E+03	4.3176E+07	7.2752E+09	1.7900E+06	1.7040E+10	1.1209E+09	2.4565E+10	5.9123E+09	1.0287E+10	7.6575E+09	1.9743E+07
CEC2017-F2	Ave	1.7132E+98	5.2459E+99	1.3942E+98	2.8180E+91	1.8297E+99	2.8919E+97	4.6577E+62	1.5007E+59	2.1132E+59	1.0000E+20	2.4429E+53	6.7940E+92
	Std	6.5535E+04	2.8733E+96	6.1034E+98	1.4676E+92	1.0016E+99	6.5535E+04	6.5535E+04	6.5535E+04	6.5535E+04	0.0000E+00	6.5535E+04	1.4631E+93
CEC2017-F3	Ave	5.4424E+05	1.1380E+05	5.8502E+05	4.2076E+05	6.4783E+05	4.4140E+05	3.2000E+05	3.4566E+05	7.7347E+05	2.8242E+05	3.3240E+05	2.6632E+05
	Std	2.6340E+05	2.8092E+04	8.2293E+04	3.7860E+04	1.2169E+05	1.3686E+05	5.5478E+04	1.1643E+04	1.1236E+05	1.7577E+04	1.4264E+04	1.7881E+04
CEC2017-F4	Ave	6.9133E+03	6.9633E+02	9.8020E+02	2.8309E+03	7.7506E+02	1.1459E+05	1.7954E+03	2.4296E+04	2.8003E+03	1.1263E+04	2.3194E+04	9.2873E+02
	Std	1.2434E+04	4.4245E+01	1.0624E+02	8.6067E+02	5.2382E+01	1.1336E+04	2.3721E+02	5.4852E+03	8.2008E+02	2.1331E+03	3.0761E+03	3.2239E+01
CEC2017-F5	Ave	1.6657E+03	1.2878E+03	1.1668E+03	1.1899E+03	1.3694E+03	2.0864E+03	1.7429E+03	1.8931E+03	1.7758E+03	1.6885E+03	1.8151E+03	1.0690E+03
	Std	2.6519E+02	6.6986E+01	8.9348E+01	7.2374E+01	4.1429E+01	3.4605E+01	5.4926E+01	8.7535E+01	1.4242E+02	5.6955E+01	5.7904E+01	4.5420E+01
CEC2017-F6	Ave	6.7421E+02	6.5607E+02	6.4701E+02	6.4818E+02	6.6369E+02	7.1106E+02	6.9351E+02	7.0109E+02	6.8271E+02	6.8710E+02	6.9496E+02	6.2828E+02
	Std	1.1894E+01	2.9144E+00	7.3504E+00	4.6355E+00	1.7247E+00	3.6568E+00	5.5434E+00	4.6054E+00	6.6987E+00	4.6228E+00	4.0835E+00	2.4833E+00
CEC2017-F7	Ave	2.8156E+03	2.9540E+03	1.8037E+03	2.4108E+03	3.2575E+03	3.9502E+03	3.7351E+03	3.8912E+03	6.4037E+03	3.5966E+03	3.2973E+03	2.1029E+03
	Std	2.2959E+02	2.0155E+02	1.3406E+02	2.2711E+02	7.9562E+01	6.2015E+01	1.1859E+02	1.0196E+02	6.1556E+02	1.1456E+02	1.2289E+02	9.4693E+01
CEC2017-F8	Ave	2.1822E+03	1.6966E+03	1.5102E+03	1.5652E+03	1.8291E+03	2.5463E+03	2.1731E+03	2.3859E+03	2.1311E+03	2.1633E+03	2.2112E+03	1.3833E+03
	Std	2.1396E+02	9.0453E+01	1.0053E+02	9.3559E+01	6.2331E+01	4.5090E+01	1.0978E+02	8.2236E+01	1.5516E+02	6.6627E+01	6.8888E+01	4.3439E+01
CEC2017-F9	Ave	6.8042E+04	2.2361E+04	4.0491E+04	5.0424E+04	2.3751E+04	8.2785E+04	5.2846E+04	6.0265E+04	4.4332E+04	4.6198E+04	7.2449E+04	1.5249E+04
	Std	1.6803E+04	1.1042E+03	8.5029E+03	6.6348E+03	2.7445E+02	4.2639E+03	5.8394E+03	4.1681E+03	5.9732E+03	7.0455E+03	4.9522E+03	1.2320E+03
CEC2017-F10	Ave	2.6312E+04	1.5355E+04	1.7792E+04	3.1382E+04	1.6843E+04	3.2902E+04	2.7016E+04	2.7334E+04	1.6259E+04	2.5017E+04	2.9920E+04	1.3832E+04
	Std	5.4735E+03	1.3807E+03	1.7501E+03	8.4885E+02	9.0378E+02	5.7391E+02	2.2921E+03	1.6001E+03	1.0401E+03	1.9119E+03	1.5764E+03	7.1653E+02
CEC2017-F11	Ave	1.5331E+05	2.3891E+03	1.2028E+04	4.4372E+04	2.3982E+04	4.0625E+05	4.2843E+04	1.6209E+05	1.2679E+05	5.5122E+04	1.2799E+05	9.9883E+03
	Std	4.4585E+04	3.1134E+02	2.3490E+03	9.1415E+03	8.1507E+03	1.6445E+05	1.5387E+04	2.2752E+04	2.8559E+04	1.2667E+04	1.5551E+04	2.3321E+03
CEC2017-F12	Ave	3.2008E+09	4.5429E+06	8.5127E+08	1.4202E+09	6.6691E+07	1.9490E+11	1.6389E+09	5.2860E+10	1.7862E+09	1.7404E+10	5.8726E+10	1.8643E+07
	Std	1.1773E+09	2.3800E+06	3.2538E+08	1.6535E+09	3.3893E+07	1.8490E+10	3.4040E+08	1.9316E+10	2.3506E+09	6.3638E+09	7.8971E+09	3.5585E+06
CEC2017-F13	Ave	1.3483E+08	9.6719E+03	1.0408E+06	2.4923E+05	3.3477E+04	4.4541E+10	3.4870E+07	8.8051E+09	2.1159E+07	8.9859E+08	9.8440E+09	3.2568E+03
	Std	1.0904E+08	7.1030E+03	3.3615E+06	4.7698E+05	1.0727E+04	6.0516E+09	2.9711E+07	4.4813E+09	8.0229E+07	5.5160E+08	2.4917E+09	5.0889E+02
CEC2017-F14	Ave	1.4795E+07	9.7513E+04	5.0287E+06	2.6422E+06	1.1918E+06	1.5375E+08	7.2412E+06	2.0206E+07	2.5964E+06	7.3815E+06	1.7691E+07	7.2234E+05
	Std	1.2972E+07	4.2834E+04	2.2676E+06	1.5004E+06	3.8870E+05	1.0607E+08	3.5462E+06	6.3584E+06	1.3462E+06	2.7645E+06	9.2974E+06	1.5964E+05
CEC2017-F15	Ave	3.6432E+07	4.7873E+03	1.5744E+05	1.0979E+04	1.8580E+04	2.4003E+10	2.9509E+06	1.9978E+09	7.4699E+07	4.2517E+07	2.4498E+09	2.0960E+03
	Std	6.0229E+07	3.0480E+03	7.3711E+04	4.8197E+03	1.3826E+04	5.0247E+09	2.2615E+06	1.6840E+09	2.9264E+08	3.1691E+07	1.2774E+09	1.9246E+02
CEC2017-F16	Ave	8.8817E+03	6.0057E+03	7.3212E+03	6.0450E+03	6.1237E+03	2.5294E+04	1.1967E+04	1.3273E+04	7.6745E+03	1.2012E+04	1.2298E+04	4.8827E+03
	Std	1.5732E+03	5.4388E+02	8.1512E+02	5.5322E+02	8.8622E+02	2.5032E+03	2.3505E+03	2.3399E+03	1.1457E+03	1.3494E+03	1.2170E+03	3.0899E+02
CEC2017-F17	Ave	8.6115E+03	5.6965E+03	5.6847E+03	5.4166E+03	5.8796E+03	1.4022E+07	8.2918E+03	1.2224E+05	6.4418E+03	7.5301E+03	3.5455E+04	4.1546E+03
	Std	1.0254E+03	5.8976E+02	6.4490E+02	7.2660E+02	8.0145E+02	1.5226E+07	1.0896E+03	1.1800E+05	6.6432E+02	1.0677E+03	2.7556E+04	2.7901E+02
CEC2017-F18	Ave	1.8516E+07	3.6427E+05	7.9899E+06	3.0602E+06	1.8895E+06	2.9652E+08	6.0773E+06	2.0003E+07	3.2473E+06	8.2509E+06	1.8330E+07	1.1908E+06
	Std	1.2223E+07	1.7419E+05	3.8223E+06	1.5694E+06	7.1396E+05	1.6321E+08	2.2448E+06	7.2124E+06	1.8659E+06	4.0101E+06	9.3858E+06	3.2142E+05
CEC2017-F19	Ave	3.7834E+07	6.6971E+03	1.0015E+07	2.0434E+04	4.0813E+04	2.4997E+10	1.1309E+07	2.1694E+09	3.9452E+07	6.2133E+07	2.4660E+09	2.1667E+03
	Std	2.8663E+07	6.9467E+03	6.4866E+06	1.7275E+04	1.8338E+05	5.3550E+09	6.3303E+06	2.8292E+09	2.1123E+08	4.8957E+07	1.1766E+09	8.7004E+01
CEC2017-F20	Ave	7.0832E+03	5.3214E+03	5.7362E+03	7.0886E+03	6.1858E+03	7.9850E+03	6.8670E+03	6.4167E+03	5.9821E+03	5.6618E+03	6.8189E+03	4.0916E+03
	Std	6.1165E+02	3.7677E+02	4.2628E+02	4.2225E+02	5.3417E+02	3.0431E+02	6.5856E+02	4.7141E+02	4.9027E+02	5.9565E+02	7.9273E+02	2.5690E+02
CEC2017-F21	Ave	3.9333E+03	3.4171E+03	3.0377E+03	3.0399E+03	3.6520E+03	4.8590E+03	4.7141E+03	4.0945E+03	4.1716E+03	3.9013E+03	3.9960E+03	2.7993E+03
	Std	1.3836E+02	1.7054E+02	9.3171E+01	1.0453E+02	2.2023E+02	1.5530E+02	2.8166E+02	1.9428E+02	2.5161E+02	1.7446E+02	8.9596E+01	3.0838E+01
CEC2017-F22	Ave	2.7274E+04	1.9370E+04	1.9648E+04	3.3465E+04	1.8920E+04	3.5346E+04	2.9693E+04	3.1076E+04	1.9285E+04	2.7957E+04	3.2381E+04	1.7051E+04
	Std	4.5762E+03	1.1735E+03	1.4358E+03	1.3332E+03	1.3232E+03	5.7492E+02	2.2482E+03	1.5258E+03	1.2452E+03	1.6235E+03	1.8328E+03	7.7487E+02
CEC2017-F23	Ave	4.8622E+03	3.8428E+03	3.5706E+03	3.7991E+03	4.1981E+03	6.3690E+03	5.9034E+03	5.4108E+03	5.0623E+03	4.6981E+03	5.5965E+03	3.2128E+03
	Std	2.6091E+02	2.0169E+02	7.7406E+01	1.0784E+02	2.0957E+02	2.1071E+02	5.0044E+02	3.6167E+02	2.7963E+02	1.2169E+02	2.7529E+02	2.7133E+01
CEC2017-F24	Ave	6.0474E+03	4.8598E+03	4.2584E+03	5.1409E+03	5.1994E+03	1.1693E+04	9.3033E+03	6.2693E+03	6.3613E+03	5.7482E+03	7.7947E+03	3.8653E+03
	Std	4.4876E+02	3.4733E+02	1.6745E+02	2.3693E+02	3.3327E+02	1.3404E+03	9.6539E+02	4.1543E+02	3.6617E+02	2.8075E+02	3.9206E+02	3.3967E+01
CEC2017-F25	Ave	6.3395E+03	3.3372E+03	3.6928E+03	5.0256E+03	3.4466E+03	2.9846E+04	4.4040E+03	1.5371E+04	4.4188E+03	8.0854E+03	1.2802E+04	3.5914E+03
	Std	3.6573E+03	6.0971E+01	9.0182E+01	5.2140E+02	5.8697E+01	1.7783E+03	2.3609E+02	1.9303E+03	6.7576E+02	9.1110E+02	1.1301E+03	3.9826E+01
CEC2017-F26	Ave	2.3080E+04	2.1829E+04	1.5541E+04	2.6015E+04	2.1353E+04	5.7365E+04	3.5399E+04	3.8982E+04	3.2976E+04	3.0194E+04	3.4265E+04	1.4427E+04
	Std	2.7695E+03	4.4402E+03	1.5883E+03	2.2593E+03	7.5145E+03	2.5656E+03	4.7589E+03	2.8668E+03	2.2870E+03	2.9804E+03	2.2204E+03	1.0678E+03
CEC2017-F27	Ave	4.5468E+03	3.7568E+03	4.0075E+03	4.7552E+03	3.8662E+03	1.4749E+04	5.5640E+03	5.7912E+03	5.6353E+03	5.1517E+03	7.7398E+03	3.6803E+03
	Std	4.2616E+02	1.2114E+02	1.8239E+02	2.4119E+02	2.6306E+02	1.1102E+03	1.2346E+03	1.0124E+03	7.2997E+02	3.8866E+02	5.7527E+02	4.0223E+01
CEC2017-F28	Ave	1.8040E+04	3.4270E+03	3.7789E+03	7.7971E+03	3.5421E+03	3.7097E+04	4.5781E+03	1.6663E+04	5.0271E+03	1.0195E+04	1.7705E+04	3.8489E+03
	Std	7.5206E+03	4.0098E+01	7.8350E+01	1.3046E+03	4.2129E+01	1.6352E+03	2.7820E+02	1.4137E+03	1.0037E+03	1.3855E+03	1.3688E+03	4.8847E+01
CEC2017-F29	Ave	1.1109E+04	7.3613E+03	9.2764E+03	8.8529E+03	7.5123E+03	1.0042E+06	1.5229E+04	2.4583E+04	1.1399E+04	1.3503E+04	1.9403E+04	6.1195E+03
	Std	1.4593E+03	7.1043E+02	9.0598E+02	7.5812E+02	6.9042E+02	4.9959E+05	1.9541E+03	9.6814E+03	1.0793E+03	1.2170E+03	5.5657E+03	3.1404E+02
CEC2017-F30	Ave	1.1358E+08	1.8219E+04	1.2176E+08	1.1559E+07	2.5928E+05	3.9497E+10	2.2493E+08	6.7101E+09	8.4726E+07	1.0296E+09	8.4744E+09	1.6474E+04
	Std	5.5941E+07	1.8578E+04	4.9197E+07	7.1841E+06	1.7823E+05	5.8794E+09	1.0004E+08	4.5735E+09	2.7473E+08	3.6580E+08	2.8481E+09	3.4078E+03

**Table 7:** Experimental results of the state-of-the-art algorithms on the CEC 2022 (dim = 20).

ID	Item	DBO	RTH	RIME	JS	SSA	BOA	HLOA	HEOA	CPSOGSA	HPHHO	AGWO	GSEA
CEC2022-F1	Ave	2.2632E+04	3.0000E+02	3.4451E+02	4.5165E+03	8.6142E+02	5.7833E+04	2.9129E+03	2.2482E+04	1.5747E+04	4.2001E+03	1.6793E+04	3.0542E+02
	Std	8.2006E+03	3.6696E-12	3.5968E+01	2.3327E+03	8.1398E+02	2.0962E+04	3.0949E+03	6.2065E+03	8.9789E+03	2.2055E+03	5.2073E+03	4.3804E+00
CEC2022-F2	Ave	4.8911E+02	4.3661E+02	4.5350E+02	4.7405E+02	4.5202E+02	3.1285E+03	4.9017E+02	6.9407E+02	4.5746E+02	5.1939E+02	6.2980E+02	4.4357E+02
	Std	4.4569E+01	2.0731E+01	1.6571E+01	3.1620E+01	1.3499E+01	7.6408E+02	4.3587E+01	1.2367E+02	1.1994E+01	4.8463E+01	7.4426E+01	4.5723E+00
CEC2022-F3	Ave	6.3271E+02	6.3528E+02	6.0145E+02	6.0497E+02	6.2393E+02	6.7804E+02	6.7103E+02	6.6721E+02	6.6340E+02	6.4706E+02	6.4551E+02	6.0002E+02
	Std	1.0491E+01	9.7860E+00	6.9795E-01	3.4140E+00	1.0302E+01	7.5421E+00	1.2067E+01	8.4464E+00	1.3623E+01	1.2419E+01	9.1849E+00	1.6808E-02
CEC2022-F4	Ave	9.0868E+02	8.7031E+02	8.5838E+02	8.4940E+02	8.9704E+02	9.7196E+02	9.0728E+02	9.2770E+02	9.1908E+02	8.9108E+02	9.0132E+02	8.2793E+02
	Std	3.1312E+01	1.6612E+01	1.8332E+01	2.2150E+01	2.1521E+01	1.4313E+01	3.5283E+01	1.9865E+01	3.2720E+01	1.3102E+01	2.0313E+01	5.4930E+00
CEC2022-F5	Ave	1.8116E+03	2.0157E+03	9.7582E+02	9.6405E+02	2.3987E+03	3.4875E+03	2.6876E+03	3.0023E+03	3.3935E+03	2.4379E+03	2.2980E+03	9.2937E+02
	Std	4.5550E+02	3.1317E+02	8.3288E+01	5.8730E+01	2.3254E+02	4.4448E+02	6.8010E+02	2.9825E+02	7.0962E+02	4.0638E+02	5.1083E+02	1.2945E+01
CEC2022-F6	Ave	4.2982E+05	5.2415E+03	7.8094E+03	4.2611E+03	7.9926E+03	1.5406E+09	7.0369E+03	1.9797E+07	5.0775E+03	3.4346E+05	1.0194E+07	1.9845E+03
	Std	1.3209E+06	3.6723E+03	5.0500E+03	3.1217E+03	7.6208E+03	9.1185E+08	5.5854E+03	3.5237E+07	3.4558E+03	4.5859E+05	1.3152E+07	9.9873E+01
CEC2022-F7	Ave	2.1248E+03	2.1496E+03	2.0616E+03	2.0485E+03	2.1268E+03	2.1956E+03	2.2925E+03	2.2216E+03	2.2217E+03	2.1270E+03	2.1698E+03	2.0300E+03
	Std	4.7496E+01	5.9726E+01	3.3797E+01	1.7062E+01	5.0252E+01	3.1332E+01	1.0567E+02	4.3500E+01	7.8271E+01	3.9048E+01	4.0362E+01	7.4381E+00
CEC2022-F8	Ave	2.2776E+03	2.3004E+03	2.2399E+03	2.2318E+03	2.2737E+03	3.6831E+03	2.5650E+03	2.2470E+03	2.3880E+03	2.2422E+03	2.2635E+03	2.2214E+03
	Std	7.1586E+01	8.4764E+01	3.7708E+01	5.0085E+00	6.3813E+01	1.4909E+03	1.9812E+02	2.9131E+01	1.2108E+02	2.2944E+01	4.8786E+01	3.1045E-01
CEC2022-F9	Ave	2.5019E+03	2.4808E+03	2.4810E+03	2.4811E+03	2.4808E+03	3.7860E+03	2.4871E+03	2.6039E+03	2.4875E+03	2.5021E+03	2.5847E+03	2.4808E+03
	Std	2.0006E+01	4.1214E-12	2.1034E-01	1.7760E+00	6.7475E-03	3.7048E+02	9.4733E+00	7.2234E+01	2.0191E+01	1.5809E+01	4.2147E+01	4.1544E-11
CEC2022-F10	Ave	3.0134E+03	4.0646E+03	2.6984E+03	2.5309E+03	3.8103E+03	3.9689E+03	5.0980E+03	4.7306E+03	4.8883E+03	2.5012E+03	4.4874E+03	2.5006E+03
	Std	8.9619E+02	9.6291E+02	2.0600E+02	7.0620E+01	7.2216E+02	2.0144E+03	8.1759E+02	1.2110E+03	7.0893E+02	2.6204E-01	1.3488E+03	5.4741E-02
CEC2022-F11	Ave	3.0960E+03	2.9133E+03	2.9196E+03	2.9163E+03	2.8907E+03	8.9661E+03	2.9888E+03	5.9755E+03	3.0732E+03	3.3935E+03	4.5286E+03	2.9000E+03
	Std	2.1675E+02	7.3030E+01	1.1331E+02	1.2685E+02	1.2427E+02	5.6268E+02	9.4961E+01	1.3525E+03	4.4038E+02	7.4606E+02	4.8452E+02	1.2582E-12
CEC2022-F12	Ave	3.0489E+03	2.9838E+03	2.9640E+03	2.9931E+03	3.0054E+03	3.3382E+03	3.2465E+03	3.0514E+03	3.2464E+03	3.0228E+03	3.1070E+03	2.9434E+03
	Std	5.6634E+01	3.8805E+01	1.8155E+01	3.1643E+01	5.8409E+01	1.1029E+02	1.5949E+02	6.4514E+01	1.6311E+02	4.7989E+01	1.0612E+02	2.8107E+00

**Table 8:** *p*-value of the state-of-the-art algorithms on the CEC 2017 (dim = 30).

Function	DBO	RTH	RIME	JS	SSA	BOA	HLOA	HEOA	CPSOGSA	HPHHO	AGWO
F1	3.0199E-11	8.8411E-07	3.0199E-11	3.0199E-11	7.0430E-07	3.0199E-11	3.0199E-11	3.0199E-11	7.0881E-08	3.0199E-11	3.0199E-11
F2	3.0199E-11	2.4386E-09	3.0199E-11	3.0199E-11	1.4643E-10	3.0199E-11	3.0199E-11	3.0199E-11	3.0199E-11	1.2118E-12	3.0199E-11
F3	3.0199E-11	3.0199E-11	9.7917E-05	3.0199E-11	3.0199E-11	3.0199E-11	8.1527E-11	3.0199E-11	3.0199E-11	3.0199E-11	3.0199E-11
F4	3.0199E-11	5.2640E-04	7.7725E-09	1.3111E-08	2.1265E-04	3.0199E-11	1.3289E-10	3.0199E-11	9.5139E-06	3.0199E-11	3.0199E-11
F5	3.0199E-11	3.0199E-11	4.0840E-05	1.1058E-04	3.0199E-11	3.0199E-11	3.0199E-11	3.0199E-11	3.0199E-11	3.0199E-11	3.0199E-11
F6	3.0199E-11	3.0199E-11	3.0199E-11	3.0199E-11	3.0199E-11	3.0199E-11	3.0199E-11	3.0199E-11	3.0199E-11	3.0199E-11	3.0199E-11
F7	3.3384E-11	3.0199E-11	7.1719E-01	3.1573E-05	3.0199E-11	3.0199E-11	3.0199E-11	3.0199E-11	3.0199E-11	3.0199E-11	3.0199E-11
F8	3.0199E-11	3.0199E-11	1.5581E-08	8.2357E-02	3.0199E-11	3.0199E-11	3.0199E-11	3.0199E-11	3.0199E-11	3.0199E-11	3.0199E-11
F9	3.0199E-11	3.0199E-11	1.8500E-08	3.1573E-05	3.0199E-11	3.0199E-11	3.0199E-11	3.0199E-11	3.0199E-11	3.0199E-11	3.0199E-11
F10	3.6897E-11	5.5727E-10	7.2884E-03	3.0199E-11	8.1014E-10	3.0199E-11	3.0199E-11	3.0199E-11	5.5727E-10	3.0199E-11	3.0199E-11
F11	3.0199E-11	3.0199E-11	3.0199E-11	4.5043E-11	3.0199E-11	3.0199E-11	3.0199E-11	3.0199E-11	3.0199E-11	3.0199E-11	3.0199E-11
F12	3.0199E-11	8.4848E-09	3.0199E-11	6.6955E-11	1.4918E-06	3.0199E-11	3.0199E-11	3.0199E-11	3.4742E-10	3.0199E-11	3.0199E-11
F13	3.0199E-11	7.7387E-06	3.0199E-11	1.1058E-04	1.6947E-09	3.0199E-11	3.0199E-11	3.0199E-11	3.0199E-11	3.0199E-11	3.0199E-11
F14	1.6132E-10	7.6950E-08	3.0199E-11	3.6459E-08	3.0199E-11	3.0199E-11	1.7769E-10	3.0199E-11	2.3715E-10	3.0199E-11	3.0199E-11
F15	3.0199E-11	1.0233E-01	5.5999E-07	1.2362E-03	4.6390E-05	3.0199E-11	3.0199E-11	3.0199E-11	8.9934E-11	3.0199E-11	3.0199E-11
F16	3.0199E-11	8.1527E-11	3.1967E-09	5.8587E-06	1.3289E-10	3.0199E-11	3.0199E-11	3.0199E-11	3.0199E-11	3.8202E-10	3.0199E-11
F17	3.0199E-11	3.0199E-11	1.2023E-08	5.2650E-05	3.0199E-11	3.0199E-11	3.0199E-11	3.0199E-11	3.0199E-11	8.1527E-11	3.0199E-11
F18	3.1589E-10	2.4327E-05	3.0199E-11	9.9186E-11	2.2273E-09	3.0199E-11	9.9186E-11	3.0199E-11	3.6897E-11	3.0199E-11	3.0199E-11
F19	3.0199E-11	1.0907E-05	8.3520E-08	7.5991E-07	1.2860E-06	3.0199E-11	3.0199E-11	3.0199E-11	1.0937E-10	3.0199E-11	3.0199E-11
F20	3.0199E-11	3.0199E-11	8.8910E-10	3.0199E-11	3.0199E-11	3.0199E-11	3.0199E-11	3.0199E-11	3.0199E-11	3.0199E-11	3.0199E-11
F21	3.0199E-11	3.0199E-11	1.2870E-09	3.1821E-04	3.0199E-11	3.0199E-11	3.0199E-11	3.0199E-11	3.0199E-11	3.0199E-11	3.0199E-11
F22	3.0199E-11	7.9555E-03	3.0199E-11	3.0199E-11	3.0199E-11	3.0199E-11	3.0199E-11	3.0199E-11	3.0199E-11	3.0199E-11	3.0199E-11
F23	3.0199E-11	3.0199E-11	9.9186E-11	3.0199E-11	3.0199E-11	3.0199E-11	3.0199E-11	3.0199E-11	3.0199E-11	3.0199E-11	3.0199E-11
F24	3.0199E-11	3.0199E-11	3.0199E-11	3.0199E-11	3.0199E-11	3.0199E-11	3.0199E-11	3.0199E-11	3.0199E-11	3.0199E-11	3.0199E-11
F25	6.6955E-11	1.8608E-06	2.1544E-10	3.0199E-11	3.1830E-03	3.0199E-11	3.0199E-11	3.0199E-11	3.0199E-11	3.0199E-11	3.0199E-11
F26	4.1997E-10	2.0054E-04	1.8682E-05	1.1711E-02	4.9980E-09	3.0199E-11	3.0199E-11	3.0199E-11	3.0199E-11	3.9648E-08	3.0199E-11
F27	3.0199E-11	2.9215E-09	3.0103E-07	3.0199E-11	3.8202E-10	3.0199E-11	3.0199E-11	3.0199E-11	3.0199E-11	3.0199E-11	3.0199E-11
F28	3.0199E-11	2.4990E-03	3.0199E-11	3.0199E-11	9.1171E-01	3.0199E-11	3.0199E-11	3.0199E-11	8.1014E-10	3.0199E-11	3.0199E-11
F29	3.0199E-11	8.1527E-11	6.0658E-11	7.3803E-10	3.3384E-11	3.0199E-11	3.0199E-11	3.0199E-11	3.0199E-11	3.0199E-11	3.0199E-11
F30	3.0199E-11	2.3168E-06	3.0199E-11	8.8910E-10	5.4617E-09	3.0199E-11	3.0199E-11	3.0199E-11	3.0199E-11	3.0199E-11	3.0199E-11

**Table 9:** *p*-value of the state-of-the-art algorithms on the CEC 2017 (dim = 50).

Function	DBO	RTH	RIME	JS	SSA	BOA	HLOA	HEOA	CPSOGSA	HPHHO	AGWO
F1	3.0199E-11	2.3768E-07	3.0199E-11	3.0199E-11	4.3531E-05	3.0199E-11	3.0199E-11	3.0199E-11	3.0199E-11	3.0199E-11	3.0199E-11
F2	3.0199E-11	3.3242E-06	3.0199E-11	3.0199E-11	4.6159E-10	3.0199E-11	3.0199E-11	3.0199E-11	3.0199E-11	1.2118E-12	3.0199E-11
F3	3.0199E-11	3.0199E-11	3.0199E-11	3.0199E-11	3.0199E-11	3.0199E-11	3.0199E-11	3.0199E-11	3.0199E-11	3.0199E-11	3.0199E-11
F4	3.0199E-11	1.1536E-01	3.0199E-11	3.0199E-11	4.1178E-06	3.0199E-11	3.0199E-11	3.0199E-11	3.0199E-11	3.0199E-11	3.0199E-11
F5	3.0199E-11	3.0199E-11	1.5292E-05	5.3221E-03	3.0199E-11	3.0199E-11	3.0199E-11	3.0199E-11	3.0199E-11	3.0199E-11	3.0199E-11
F6	3.0199E-11	3.0199E-11	3.0199E-11	3.0199E-11	3.0199E-11	3.0199E-11	3.0199E-11	3.0199E-11	3.0199E-11	3.0199E-11	3.0199E-11
F7	3.0199E-11	3.0199E-11	9.7052E-01	7.0881E-08	3.0199E-11	3.0199E-11	3.0199E-11	3.0199E-11	3.0199E-11	3.0199E-11	3.0199E-11
F8	3.0199E-11	6.0658E-11	2.2658E-03	1.7666E-03	3.0199E-11	3.0199E-11	3.0199E-11	3.0199E-11	3.0199E-11	3.0199E-11	3.0199E-11
F9	3.0199E-11	3.0199E-11	1.2870E-09	3.0199E-11	3.0199E-11	3.0199E-11	3.0199E-11	3.0199E-11	3.0199E-11	3.0199E-11	3.0199E-11
F10	3.0199E-11	1.4110E-09	5.5727E-10	3.0199E-11	3.0199E-11	3.0199E-11	3.0199E-11	3.0199E-11	1.7769E-10	3.0199E-11	3.0199E-11
F11	3.0199E-11	8.1527E-11	3.0199E-11	3.0199E-11	1.2057E-10	3.0199E-11	3.0199E-11	3.0199E-11	3.0199E-11	3.0199E-11	3.0199E-11
F12	3.0199E-11	2.1947E-08	3.0199E-11	3.0199E-11	1.4110E-09	3.0199E-11	3.0199E-11	3.0199E-11	3.0199E-11	3.0199E-11	3.0199E-11
F13	3.0199E-11	4.0772E-11	3.0199E-11	3.0199E-11	3.0199E-11	3.0199E-11	3.0199E-11	3.0199E-11	3.0199E-11	3.0199E-11	3.0199E-11
F14	3.0199E-11	5.1857E-07	8.1527E-11	3.9648E-08	1.8567E-09	3.0199E-11	3.0199E-11	3.0199E-11	3.0199E-11	3.0199E-11	3.0199E-11
F15	3.0199E-11	1.0105E-08	3.0199E-11	6.7362E-06	1.9568E-10	3.0199E-11	3.0199E-11	3.0199E-11	3.0199E-11	3.0199E-11	3.0199E-11
F16	3.0199E-11	8.1527E-11	3.1589E-10	2.5721E-07	3.8202E-10	3.0199E-11	3.0199E-11	3.0199E-11	3.0199E-11	3.0199E-11	3.0199E-11
F17	3.0199E-11	3.0199E-11	3.0199E-11	4.1825E-09	3.0199E-11	3.0199E-11	3.0199E-11	3.0199E-11	3.0199E-11	3.0199E-11	3.0199E-11
F18	4.0772E-11	2.3715E-10	1.3289E-10	3.6459E-08	4.1997E-10	3.0199E-11	8.4848E-09	3.0199E-11	2.2273E-09	3.0199E-11	3.0199E-11
F19	3.3384E-11	7.8446E-01	1.2541E-07	3.6439E-02	9.9410E-01	3.0199E-11	3.0199E-11	3.0199E-11	7.7387E-06	3.0199E-11	3.0199E-11
F20	3.0199E-11	3.0199E-11	3.8202E-10	4.5726E-09	4.5043E-11	3.0199E-11	3.0199E-11	3.0199E-11	3.0199E-11	3.0199E-11	3.0199E-11
F21	3.0199E-11	1.7769E-10	3.0199E-11	3.5638E-04	3.0199E-11	3.0199E-11	3.0199E-11	3.0199E-11	3.0199E-11	3.0199E-11	3.0199E-11
F22	3.0199E-11	2.3715E-10	1.8500E-08	3.1967E-09	1.0937E-10	3.0199E-11	3.0199E-11	3.0199E-11	2.6099E-10	3.0199E-11	3.0199E-11
F23	3.0199E-11	3.0199E-11	6.6955E-11	3.0199E-11	3.0199E-11	3.0199E-11	3.0199E-11	3.0199E-11	3.0199E-11	3.0199E-11	3.0199E-11
F24	3.0199E-11	3.0199E-11	4.5043E-11	3.0199E-11	3.0199E-11	3.0199E-11	3.0199E-11	3.0199E-11	3.0199E-11	3.0199E-11	3.0199E-11
F25	3.0199E-11	9.5139E-06	5.5611E-04	3.0199E-11	3.1830E-01	3.0199E-11	3.0199E-11	3.0199E-11	3.0199E-11	3.0199E-11	3.0199E-11
F26	7.1186E-09	1.1077E-06	2.5188E-01	3.0199E-11	1.0000E+00	3.0199E-11	3.0199E-11	3.0199E-11	3.0199E-11	5.0723E-10	3.0199E-11
F27	3.0199E-11	3.8249E-09	2.2273E-09	3.0199E-11	1.4110E-09	3.0199E-11	3.0199E-11	3.0199E-11	3.0199E-11	3.0199E-11	3.0199E-11
F28	3.0199E-11	1.0105E-08	3.8349E-06	3.0199E-11	8.1200E-04	3.0199E-11	3.0199E-11	3.0199E-11	3.0199E-11	3.0199E-11	3.0199E-11
F29	3.0199E-11	4.9752E-11	3.0199E-11	3.0199E-11	3.0199E-11	3.0199E-11	3.0199E-11	3.0199E-11	3.0199E-11	3.0199E-11	3.0199E-11
F30	3.0199E-11	1.6798E-03	3.0199E-11	3.0199E-11	3.4742E-10	3.0199E-11	3.0199E-11	3.0199E-11	3.0199E-11	3.0199E-11	3.0199E-11

**Table 10:** *p*-value of the state-of-the-art algorithms on the CEC 2017 (dim = 100).

Function	DBO	RTH	RIME	JS	SSA	BOA	HLOA	HEOA	CPSOGSA	HPHHO	AGWO
F1	3.0199E-11	3.0199E-11	3.6897E-11	3.0199E-11	3.0199E-11	3.0199E-11	3.0199E-11	3.0199E-11	3.0199E-11	3.0199E-11	3.0199E-11
F2	3.0199E-11	1.6813E-04	8.9934E-11	3.0199E-11	3.1967E-09	3.0199E-11	3.0199E-11	3.0199E-11	3.0199E-11	1.2118E-12	3.0199E-11
F3	3.0199E-11	3.0199E-11	3.0199E-11	3.0199E-11	3.0199E-11	3.0199E-11	3.5708E-06	3.0199E-11	3.0199E-11	2.0523E-03	3.0199E-11
F4	3.0199E-11	3.0199E-11	3.6439E-02	3.0199E-11	3.6897E-11	3.0199E-11	3.0199E-11	3.0199E-11	3.0199E-11	3.0199E-11	3.0199E-11
F5	3.0199E-11	3.0199E-11	4.8011E-07	6.5183E-09	3.0199E-11	3.0199E-11	3.0199E-11	3.0199E-11	3.0199E-11	3.0199E-11	3.0199E-11
F6	3.0199E-11	3.0199E-11	3.0199E-11	3.0199E-11	3.0199E-11	3.0199E-11	3.0199E-11	3.0199E-11	3.0199E-11	3.0199E-11	3.0199E-11
F7	3.0199E-11	3.0199E-11	1.5465E-09	3.0811E-08	3.0199E-11	3.0199E-11	3.0199E-11	3.0199E-11	3.0199E-11	3.0199E-11	3.0199E-11
F8	3.0199E-11	7.3891E-11	1.2860E-06	4.5043E-11	3.0199E-11	3.0199E-11	3.0199E-11	3.0199E-11	3.0199E-11	3.0199E-11	3.0199E-11
F9	3.0199E-11	3.0199E-11	3.0199E-11	3.0199E-11	3.0199E-11	3.0199E-11	3.0199E-11	3.0199E-11	3.0199E-11	3.0199E-11	3.0199E-11
F10	3.0199E-11	1.6351E-05	3.0199E-11	3.0199E-11	3.0199E-11	3.0199E-11	3.0199E-11	3.0199E-11	2.3715E-10	3.0199E-11	3.0199E-11
F11	3.0199E-11	3.0199E-11	3.1830E-03	3.0199E-11	9.9186E-11	3.0199E-11	3.0199E-11	3.0199E-11	3.0199E-11	3.0199E-11	3.0199E-11
F12	3.0199E-11	3.0199E-11	3.0199E-11	3.0199E-11	3.0199E-11	3.0199E-11	3.0199E-11	3.0199E-11	3.0199E-11	3.0199E-11	3.0199E-11
F13	3.0199E-11	4.9752E-11	3.0199E-11	3.0199E-11	3.0199E-11	3.0199E-11	3.0199E-11	3.0199E-11	3.0199E-11	3.0199E-11	3.0199E-11
F14	3.0199E-11	3.0199E-11	3.0199E-11	3.0199E-11	2.3168E-06	3.0199E-11	3.0199E-11	3.0199E-11	7.1186E-09	3.0199E-11	3.0199E-11
F15	3.0199E-11	3.4971E-09	3.0199E-11	3.0199E-11	3.0199E-11	3.0199E-11	3.0199E-11	3.0199E-11	3.0199E-11	3.0199E-11	3.0199E-11
F16	3.0199E-11	5.5727E-10	3.0199E-11	1.6132E-10	3.3520E-08	3.0199E-11	3.0199E-11	3.0199E-11	3.0199E-11	3.0199E-11	3.0199E-11
F17	3.0199E-11	3.0199E-11	3.0199E-11	7.3803E-10	3.0199E-11	3.0199E-11	3.0199E-11	3.0199E-11	3.0199E-11	3.0199E-11	3.0199E-11
F18	3.0199E-11	1.0937E-10	3.0199E-11	5.4617E-09	4.2175E-04	3.0199E-11	3.0199E-11	3.0199E-11	1.8500E-08	3.0199E-11	3.0199E-11
F19	3.0199E-11	6.6955E-11	3.0199E-11	3.0199E-11	3.0199E-11	3.0199E-11	3.0199E-11	3.0199E-11	3.0199E-11	3.0199E-11	3.0199E-11
F20	3.0199E-11	3.0199E-11	3.0199E-11	3.0199E-11	3.0199E-11	3.0199E-11	3.0199E-11	3.0199E-11	3.0199E-11	3.0199E-11	3.0199E-11
F21	3.0199E-11	3.0199E-11	3.0199E-11	3.0199E-11	3.0199E-11	3.0199E-11	3.0199E-11	3.0199E-11	3.0199E-11	3.0199E-11	3.0199E-11
F22	3.0199E-11	2.9215E-09	3.4971E-09	3.0199E-11	1.4733E-07	3.0199E-11	3.0199E-11	3.0199E-11	4.9980E-09	3.0199E-11	3.0199E-11
F23	3.0199E-11	3.0199E-11	3.0199E-11	3.0199E-11	3.0199E-11	3.0199E-11	3.0199E-11	3.0199E-11	3.0199E-11	3.0199E-11	3.0199E-11
F24	3.0199E-11	3.0199E-11	3.0199E-11	3.0199E-11	3.0199E-11	3.0199E-11	3.0199E-11	3.0199E-11	3.0199E-11	3.0199E-11	3.0199E-11
F25	3.0199E-11	3.0199E-11	1.7479E-05	3.0199E-11	2.3715E-10	3.0199E-11	3.0199E-11	3.0199E-11	3.0199E-11	3.0199E-11	3.0199E-11
F26	3.0199E-11	5.5727E-10	2.7548E-03	3.0199E-11	1.1077E-06	3.0199E-11	3.0199E-11	3.0199E-11	3.0199E-11	3.0199E-11	3.0199E-11
F27	3.8202E-10	7.9590E-03	9.9186E-11	3.0199E-11	4.2259E-03	3.0199E-11	3.0199E-11	3.0199E-11	3.0199E-11	3.0199E-11	3.0199E-11
F28	3.0199E-11	3.0199E-11	3.3681E-05	3.0199E-11	3.0199E-11	3.0199E-11	3.0199E-11	3.0199E-11	3.0199E-11	3.0199E-11	3.0199E-11
F29	3.0199E-11	1.4110E-09	3.0199E-11	3.0199E-11	2.8716E-10	3.0199E-11	3.0199E-11	3.0199E-11	3.0199E-11	3.0199E-11	3.0199E-11
F30	3.0199E-11	8.7710E-02	3.0199E-11	3.0199E-11	3.0199E-11	3.0199E-11	3.0199E-11	3.0199E-11	3.0199E-11	3.0199E-11	3.0199E-11

**Table 11:** *p*-value of the state-of-the-art algorithms on the CEC 2022 (dim = 20).

Function	DBO	RTH	RIME	JS	SSA	BOA	HLOA	HEOA	CPSOGSA	HPHHO	AGWO
F1	3.0199E-11	2.9543E-11	2.8716E-10	3.0199E-11	5.0723E-10	3.0199E-11	3.0199E-11	3.0199E-11	3.0199E-11	3.0199E-11	3.0199E-11
F2	3.0199E-11	5.5498E-02	9.2603E-09	3.3384E-11	7.1186E-09	3.0199E-11	5.4617E-09	3.0199E-11	3.0199E-11	3.0199E-11	3.0199E-11
F3	3.0199E-11	3.0199E-11	3.0199E-11	3.0199E-11	3.0199E-11	3.0199E-11	3.0199E-11	3.0199E-11	3.0199E-11	3.0199E-11	3.0199E-11
F4	3.0180E-11	3.0180E-11	2.8700E-10	3.8297E-05	3.0180E-11	3.0180E-11	3.0180E-11	3.0180E-11	3.0180E-11	3.0180E-11	3.0180E-11
F5	3.0199E-11	3.0199E-11	4.0330E-03	2.1566E-03	3.0199E-11	3.0199E-11	3.0199E-11	3.0199E-11	3.0199E-11	3.0199E-11	3.0199E-11
F6	3.0199E-11	1.0666E-07	3.0199E-11	3.5923E-05	6.0459E-07	3.0199E-11	7.3803E-10	3.0199E-11	5.4617E-09	3.0199E-11	3.0199E-11
F7	2.6099E-10	3.0199E-11	5.5999E-07	4.4205E-06	3.0199E-11	3.0199E-11	3.0199E-11	3.0199E-11	3.0199E-11	3.0199E-11	3.0199E-11
F8	3.0199E-11	3.0199E-11	3.0199E-11	3.0199E-11	2.3715E-10	3.0199E-11	3.0199E-11	3.0199E-11	3.0199E-11	3.0199E-11	3.0199E-11
F9	3.0104E-11	4.0950E-11	3.0104E-11	3.0104E-11	1.3140E-01	3.0104E-11	3.0104E-11	3.0104E-11	3.0104E-11	3.0104E-11	3.0104E-11
F10	8.1527E-11	3.0199E-11	5.5999E-07	3.3384E-11	5.5727E-10	3.0199E-11	3.0199E-11	3.0199E-11	3.0199E-11	3.0199E-11	3.0199E-11
F11	1.5693E-11	3.0236E-09	5.1735E-09	5.1735E-09	7.7464E-07	1.5693E-11	3.1458E-10	1.5693E-11	1.5693E-11	3.1458E-10	1.5693E-11
F12	3.0199E-11	1.4643E-10	3.6459E-08	3.0199E-11	4.5043E-11	3.0199E-11	3.0199E-11	3.0199E-11	3.0199E-11	3.0199E-11	3.0199E-11

**Table 12:** Friedman mean rank test for the state-of-the-art algorithms.

Suites	CEC2017						CEC2022	
Dimensions	30		50		100		20	
Algorithms	M.R	T.R	M.R	T.R	M.R	T.R	M.R	T.R
DBO	7.60	8	7.60	8	7.73	9	7.25	7
RTH	3.17	2	2.93	2	2.27	2	3.92	4
RIME	3.77	4	3.67	3	4.13	4	3.58	3
JS	3.60	3	4.40	5	5.37	5	3.50	2
SSA	4.60	5	4.30	4	3.97	3	4.75	5
BOA	11.83	12	11.93	12	11.80	12	11.42	12
HLOA	8.70	9	8.37	9	7.67	8	8.75	9
HEOA	10.07	11	10.07	11	9.93	11	9.92	11
CPSOGSA	7.43	7	6.97	6	6.67	6	8.33	8
HPHHO	7.10	6	7.03	7	7.17	7	6.58	6
AGWO	8.90	10	9.40	10	9.70	10	8.83	10
GSEA	1.23	1	1.33	1	1.60	1	1.17	1

## Data Availability

All data in this paper are included in the manuscript.
